# Longitudinal Neuroimaging in Pediatric Traumatic Brain Injury: Current State and Consideration of Factors That Influence Recovery

**DOI:** 10.3389/fneur.2019.01296

**Published:** 2019-12-13

**Authors:** Hannah M. Lindsey, Elisabeth A. Wilde, Karen Caeyenberghs, Emily L. Dennis

**Affiliations:** ^1^Department of Neurology, University of Utah, Salt Lake City, UT, United States; ^2^Department of Psychology, Brigham Young University, Provo, UT, United States; ^3^Department of Physical Medicine and Rehabilitation, Baylor College of Medicine, Houston, TX, United States; ^4^Cognitive Neuroscience Unit, School of Psychology, Deakin University, Burwood, VIC, Australia

**Keywords:** traumatic brain injury, pediatric, neuroimaging, longitudinal, brain development, neuroplasticity

## Abstract

Traumatic brain injury (TBI) is a leading cause of death and disability for children and adolescents in the U.S. and other developed and developing countries. Injury to the immature brain varies greatly from that of the mature, adult brain due to numerous developmental, pre-injury, and injury-related factors that work together to influence the trajectory of recovery during the course of typical brain development. Substantial damage to brain structure often underlies subsequent functional limitations that persist for years following pediatric TBI. Advances in neuroimaging have established an important role in the acute management of pediatric TBI, and magnetic resonance imaging (MRI) techniques have a particular relevance for the sequential assessment of long-term consequences from injuries sustained to the developing brain. The present paper will discuss the various factors that influence recovery and review the findings from the present neuroimaging literature to assess altered development and long-term outcome following pediatric TBI. Four MR-based neuroimaging modalities have been used to examine recovery from pediatric TBI longitudinally: (1) T_1_-weighted structural MRI is sensitive to morphological changes in gray matter volume and cortical thickness, (2) diffusion-weighted MRI is sensitive to changes in the microstructural integrity of white matter, (3) MR spectroscopy provides a sensitive assessment of metabolic and neurochemical alterations in the brain, and (4) functional MRI provides insight into the functional changes that occur as a result of structural damage and typical developmental processes. As reviewed in this paper, 13 cohorts have contributed to only 20 studies published to date using neuroimaging to examine longitudinal changes after TBI in pediatric patients. The results of these studies demonstrate considerable heterogeneity in post-injury outcome; however, the existing literature consistently shows that alterations in brain structure, function, and metabolism can persist for an extended period of time post-injury. With larger sample sizes and multi-site cooperation, future studies will be able to further examine potential moderators of outcome, such as the developmental, pre-injury, and injury-related factors discussed in the present review.

## Introduction

Recent estimates suggest that a child under the age of 14 sustains a traumatic brain injury (TBI) every 60 s in the United States (1). While TBI-related deaths in children have substantially decreased over the past decade, TBI remains the leading cause of death among children and adolescents (2). Of the survivors, ~62% of children who sustained moderate-to-severe injuries, and 14% of those with milder injuries suffer from long-term disability (3). The trajectory of recovery from an injury sustained to the developing brain differs greatly from that of the mature, adult brain (4, 5). Thus, an understanding of the long-term effects of early brain injury on subsequent neurodevelopment is vital for the accurate understanding and prediction of a child's outcome and recovery.

Currently, falls, sports- and recreation-related blunt force trauma, and motor vehicle accidents are the leading causes of pediatric TBI (1, 3). These mechanisms commonly give rise to acceleration-deceleration injuries that result in diffuse axonal injury (DAI), which refers to the extensive structural damage that occurs to otherwise highly organized neural tissue due to the abrupt stretching, twisting, and shearing of axons in the event of a mechanical blow. DAI is critically related to functional outcomes following early brain injury, as it leads to reductions in white matter integrity, disrupting the connectivity of the neural networks that give rise to behavioral and cognitive function (6). Plasticity moves anteriorly during typical neural development, where the frontal and temporal regions of the brain are among the last to develop (7, 8). Due to the close proximity of these brain regions to the bony structure of the anterior and middle fossa of the skull (9), they are the most vulnerable in acceleration-deceleration injuries. For this reason, early brain insult likely affects the maturation of the frontal and temporal cortices, as well as the white matter pathways connecting them to other areas of the brain. Such disruption is known to have detrimental and long-term consequences on the development of critical neurobehavioral functions localized within these regions, such as executive function (10, 11), learning and memory (12), emotional control (13), behavioral self-regulation (14), and social adaptive behavior (15).

**Figure 1:**
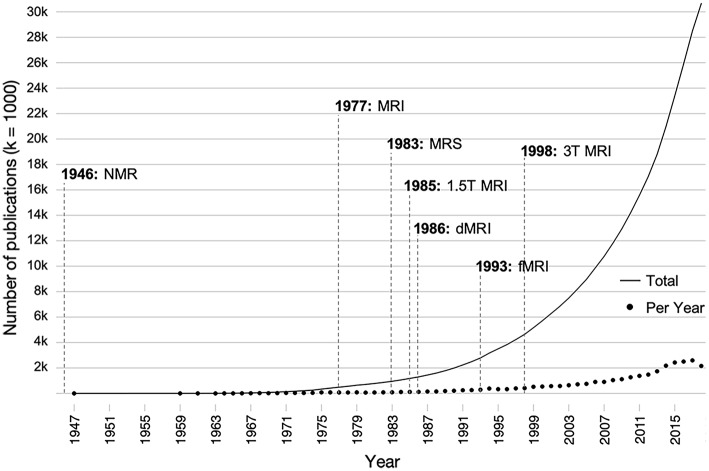
Influence of the development of various magnetic resonance-based imaging modalities on the number of publications on brain development over time. Individual points indicate the number of papers published per a given year, and the solid line indicates the total number of publications over all time. Publications were found using the search terms (brain AND development OR neurodevelopment) AND (childhood OR adolescent OR pediatric) AND (structure OR function) in PubMed. dMRI, diffusion magnetic resonance imaging; fMRI, functional magnetic resonance imaging; MRI, magnetic resonance imaging; MRS, magnetic resonance spectroscopy; NMR, nuclear magnetic resonance; T, tesla.

Despite the fact that injury to the developing brain has potentially more devastating long-term consequences than injury to the adult brain (16), there is substantially less literature on the long-term consequences of pediatric TBI compared to adults with TBI. In particular, relatively few studies have utilized these MRI methods for the longitudinal assessment of recovery from early brain injury. In addition to the great number of developmental, pre-injury, and injury-related factors that influence outcome after pediatric TBI (17), a major reason for the lack of longitudinal research lies in the substantial cost associated with clinical imaging. Additionally, sensitive measurement tools that are suitable for the prediction of outcome in a pediatric population have only recently become available. The literature on early brain development has increased 10-fold over the last 25 years as technological advances have been made in neuroimaging techniques, specifically those involving the use of magnetic resonance imaging (MRI; see Figure 1). Likewise, advances in the field of neuroimaging have established an important role in identifying the sequelae and determining the acute management of pediatric TBI over the last two decades (18).

Although computed tomography (CT) is necessary for the rapid evaluation of primary head trauma complications that require immediate intervention (e.g., extra-axial hemorrhage, skull fracture, etc.), its clinical utility beyond this is generally somewhat limited, as the extent of axonal damage due to DAI is commonly underestimated with CT. Structural MRI is not only more sensitive to identifying DAI than CT, but it also has a particular relevance for the sequential assessment of the longitudinal consequences of brain injury (19). While structural MRI (sMRI) has greater prognostic utility in TBI than CT, it is unable to fully account for the complexity of pediatric TBI neuropathology when only used in the first weeks following the injury (20). Serial volumetric analysis, however, can provide insight into long-term neurodegeneration that occurs as a result of ongoing secondary injury pathology. More advanced techniques, such as diffusion-weighted MRI (dMRI), magnetic resonance spectroscopy (MRS), and functional MRI (fMRI) have greater sensitivity to the primary and secondary injuries after TBI, therefore establishing increased value for predicting long-term outcome after injuries are sustained to the developing brain (21, 22). Diffusion tensor imaging (DTI) is a dMRI technique that is sensitive to the long-term pathological effects of DAI on the microstructural integrity of white matter (23, 24); however, DTI is unable to reveal the underlying processes for such effects. MRS allows for the non-invasive measurement of metabolites in the brain, which vary by anatomic region and change rapidly as the brain develops through the adolescent years. Through the examination of intracellular metabolic status, MRS is able to detect several metabolites that are sensitive to the pathology associated with secondary brain injury cascades and is therefore capable of providing direct evidence of microscopic neuronal injury. Substantial damage to brain structure can occur in pediatric TBI, and such damage often underlies subsequent functional limitations in physical, emotional, cognitive, behavioral, adaptive, and academic abilities that persist for years following the injury (25). Adults who suffered from childhood TBI 15-years prior report significantly poorer perceptions of their health-related quality of life due to ongoing functional limitations, regardless of injury severity (26). There is an intimate relationship between brain structure and function, and this complimentary relationship extends toward the use of functional and structural neuroimaging modalities in the evaluation of long-term outcome following brain injury.

Prior to our review of the present longitudinal neuroimaging literature, we will discuss several developmental, injury-related, and pre-injury factors that are known to influence development and recovery from damage to an immature brain. We believe that such a discussion is vital, as the field currently lacks a complete understanding of the way in which these factors interact with each other to further complicate the trajectory of recovery in the presence of injury-induced alterations to brain development. The complex interaction that occurs among such influences may have unpredictable negative consequences for otherwise adaptive structural and functional neuroplasticity that occurs in response to tissue damage (5). While there is an appreciation in the field for the importance of considering such factors in relation to planning rehabilitation and estimating the overall recovery period these factors are less often included in neuroimaging analyses. There is considerable heterogeneity post-injury, which can lead to inconsistent results in the neuroimaging literature. Understanding the ways in which developmental, injury-related, and pre-injury factors may impact neuroplasticity in the developing brain may be key to explaining more of this variance.

Following this discussion, we will briefly review the findings from longitudinal studies employing MR-based neuroimaging modalities to increase our current understanding of altered development and long-term outcome following pediatric TBI. Recent reviews of studies utilizing various neuroimaging modalities in children with moderate-to-severe TBI [msTBI; (27)] and of studies utilizing sMRI (28) or dMRI (29) to evaluate outcome from mild-to-severe pediatric TBI have been published. However, no reviews of the current literature, have focused solely on longitudinal neuroimaging studies (i.e., imaging at least two points in time) using various MR-based modalities to characterize outcome following mild-to-severe pediatric TBI; henceforth, such studies will be the focus of the present review. Furthermore, we will discuss whether the developmental, injury-related, and pre-injury factors known to influence plasticity and recovery from early TBI are considered in analyses conducted by the longitudinal studies reviewed here. Finally, we will conclude with a discussion of the current gaps in the literature and provide suggestions for future directions that should be taken in the field.

## Factors That Influence Plasticity and Recovery

An important distinction between pediatric and adult TBI is that the primary cause of pediatric injuries vary significantly by age group and can present in various ways (1, 30, 31). Various types of head injuries (e.g., blunt, penetrating, acceleration/deceleration) are closely related to the circumstantial mechanisms that caused them (32), yet great heterogeneity exists in the clinical presentation manifested by TBI, suggesting that similar heterogeneity exists in the underlying pathological features of the damaged brain. No two brain injuries are equal due to the complex interaction of inter-individual differences in the timing and circumstances in which the injury occurred, the severity, biomechanics, and nature of the injury itself, and intra-individual factors such as age, sex, and quality of the pre-injury environment (33–36). For this reason, it is essential that such factors are considered in the assessment of outcome and prediction of recovery following pediatric brain injury.

### Developmental Factors

The central nervous system is inherently plastic in its capacity to respond to the environment and experience in a dynamic manner through modification of its neural circuitry (37). The phenomenological nature of neuroplasticity is linked to the development of the brain and function across the lifespan, and it is a beneficial property in the context of healthy development (17, 38). In the context of early brain injury, however, the influence of plasticity on brain development may be detrimental, as the interrupted developmental processes can be altered permanently or cease entirely (39, 40).

#### Age at Injury

A major difference between a mature and a developing brain is the presumed capacity for heightened plasticity in the latter (40, 41). Historically, research of the effects that age at the time of injury has on outcome suggests that worse cognitive outcomes are associated with a younger age when injury occurred, and injuries sustained before age eight were believed to have the worst prognosis (33, 42, 43). More recently, a complex, non-linear relationship between age at injury and cognitive outcome has been demonstrated in the literature (17, 44, 45). Developmental research suggests that there are critical periods for the acquisition of specific skill sets (46). A critical period in development designates a maximally-sensitive, developmental phase of enhanced experience-expectant plasticity when the brain is heavily influenced by environmental demands (37). During critical periods, the brain undergoes significant structural and functional growth as it learns, adapts, and makes connections with other parts of the brain. This heightened sensitivity, however, simultaneously increases the brain's vulnerability to disruption in the environment, therefore heightening its susceptibility to insult. Brain damage that occurs during a critical period can have a more profound effect on skill acquisition relative to the effects of injuries that occur during non-critical periods (42, 47), as predetermined developmental processes are derailed, natural resources are depleted, and the typical developmental course that guides recovery is no longer available (48, 49). The timing of a brain injury is therefore important to consider, because injuries that occur during critical brain development periods tend to result in more extensive damage to whatever region is currently undergoing accelerated maturation, and subsequently, greater deficits can occur in whatever functions are to be localized in that region (50). An in-depth examination of the impact of age-at-injury on brain maturation is needed, however, as “critical periods” for injury likely depend on the outcome measure used (e.g., language function vs. executive functions). Longitudinal studies suggest that the cognitive skills of children with early brain insults (before age 10) tend to develop slower than that of non-brain injured children (17, 50), due to a heightened vulnerability in skill acquisition during critical periods (51, 52).

#### Time Since Injury

Time since injury is also an important factor to consider, as atypical timing of neural development may result in progressive functional deterioration that occurs due to a child's inability to effectively interact with the environment (42). Alternatively, the child may grow into the deficit in later years, where certain functional deficits do not emerge until the child reaches the appropriate stage of development for some skill to develop (17, 53–55). For example, higher-order executive functions develop later in adolescence and deficits may not be evident until the child reaches the appropriate age at which those functions typically emerge. Over time, dysfunction can become more apparent as the child grows into the deficit and fails to acquire the same skills his or her peers are developing. The result is arrested functional maturation over time, in which deficits become more apparent as the child ages (17, 53, 56).

### Injury-Related Factors

#### Severity of the Injury

More severe injuries are associated with worse physical and cognitive performance in the subacute (≤7 days post-injury), acute (≤90 days post-injury), and chronic (>90 days post-injury) periods of recovery from pediatric TBI (57). TBI severity is typically categorized as mild, moderate, or severe based on the patient's initial clinical presentation, and the primary measures used to classify injury severity in children include the Glasgow Coma Scale [GCS; (58)] and the Pediatric Coma Scale (59), where scores between 13–15, 9–12, and 3–8 indicate mild, moderate, and severe injuries, respectively. Complicated mild TBI is sometimes used to designate the severity of an injury when GCS is between 13 and 15 but abnormal day-of-injury imaging results are present [e.g., skull fracture, intracranial lesion; (60, 61)]. Other common severity indices include the duration of posttraumatic amnesia, the duration of loss of consciousness or coma, and the length of hospital stay. In a large study of 2,940 children who sought medical treatment for TBI, 84.5% suffered from mild injuries, 13.2% suffered from moderate injuries, and 2.3% suffered from severe injuries (62). The results of a meta-analysis (63) demonstrated no statistically significant effects on overall neurocognitive outcome after mild TBI (mTBI), whereas children with complicated mTBI and msTBI have been shown to recover at slower rates and have poor cognitive outcomes up to several years post-injury (44, 60, 64).

#### Lesion Characteristics

The heterogeneity of various biomechanical, pathological, and physiological mechanisms of primary and secondary injuries are reflected by structural imaging abnormalities observed in chronic TBI [for a review, see (19, 65)]. Size, laterality, location, and extent of the lesion can all impact post-injury outcome. Poorer outcomes after pediatric TBI have consistently been seen in patients with larger, more diffuse, and bilateral injuries (66). Smaller, unilateral lesions tend to demonstrate the greatest plasticity, resulting in relatively good recovery (67, 68), and it is suggested that such focal damage forces the interhemispheric transfer of function, resulting in minimal impact on functional abilities (69). Other studies investigating laterality have reported that right vs. left hemisphere lesions to the frontal lobe lead to somewhat different impairment profiles across cognitive domains (53, 56, 70, 71). In contrast to that of adult TBI, the relationship between lesion location and outcome following pediatric TBI is not consistently documented (72). Intact (i.e., undamaged) frontal and parietal cortices are important for functional reorganization or the recruitment of additional cerebral regions to compensate for the functions localized in the damaged region (73); however functional outcome has not been linked to functional reorganization, *per se* (19, 74, 75). It is possible that incomplete functional localization of the immature brain during early childhood leads to a less severe impact of lesion location or laterality on recovery. Rather, the extent of damaged tissue is the strongest predictor of outcome, suggesting that the integrity of the whole brain network may be necessary for efficient functioning in children (17).

#### Mechanism of Injury

**Figure 2:**
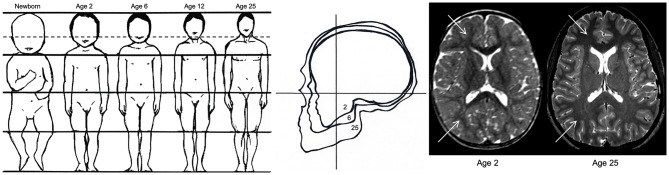
Anthropometric differences between children and adults. The image on the left demonstrates the decreasing ratio of head-to-body size from birth to adulthood, which increases the likelihood of traumatic brain injury in children relative to adults. The image in the middle reflects the increasing ratio between facial and cranial size between ages 2, 6, and 25, demonstrating the greater risk for skull trauma in children relative to adults. The image on the right reflects the difference in T_2_-weighted contrast hyperintensity (indicated by white arrows) due to less myelination and a greater concentration of water in a 2-year-old brain relative to a fully developed 25-year-old brain. The immaturity of the white matter in the newborn makes the brain “softer” and more prone to acceleration-deceleration injury. Adapted from Pinto et al. (78).

Unintentional blunt force trauma to the head involves contact forces that produce focal lacerations, fractures, and contusions to the brain, scalp, and skull, and often results in epidural hemorrhages. Acceleration-deceleration injuries involve inertial forces, which cause excessive movement of the brain, yielding more diffuse injuries such as concussion, subdural hematoma, and diffuse vascular damage (76, 77). Anthropometric development and age-specific biomechanical properties of a growing child's skull, face, brain, and neck muscles make children more or less susceptible to specific injuries that are less often or not seen in adults [see Figure 2; (78)]. Children have a greater head-to-body ratio, which increases the probability that damage will occur to the head in the event of trauma. Furthermore, a greater head-to-body ratio contributes to the relative weight of the head compared to the rest of the body, which results in different dynamics of head acceleration between children and adults. Relative to adults, children also have a greater relative proportion of cerebral blood volume and water content due to the degree of myelination that has occurred, making the brain softer and more susceptible to acceleration-deceleration injury. Because a child's brain tissue, skull, and neck musculature is not fully developed, they are more susceptible to posttraumatic edema, ischemic insult, and DAI when exposed to the inertial forces and direct blows associated with falls, sports-related injuries, and motor vehicle accidents, which are the most common mechanisms of injury in children (22, 35).

### Pre-injury Factors

#### Sex

Biological sex is likely to affect neurodevelopment after early brain injury, and MRI research in typical human development demonstrated differential rates of cortical development in males and females, where gray matter density peaks around age 10 in females but not until age 12 in males (7, 79, 80). There is also evidence for greater dendritic volume in the left hemisphere (81) and increased bilateral cortical activation (82) in the young female brain. The animal literature suggests that sex-related differences in the development of gray and white matter is influenced by endogenous hormones, specifically the increased progesterone levels in females. Research using rodent TBI-models report that females recover better than males [for a review, see (83)], and many studies have provided evidence for the neuroprotective effects of progesterone against secondary mechanisms of brain injury, although results have been somewhat conflicting. Increased levels of progesterone have been shown to reduce brain edema, increase neuronal survival, and impact the expression of genes involved in the regulation of inflammatory responses and apoptosis in brain injured rats (83, 84). Progesterone has also been implicated as a promotor of axon regeneration and remyelination (85, 86), and this is supported by research demonstrating sex differences in neuroplasticity following early brain injury to rats (87).

In human research, biological sex plays an important role in psychosocial development, where females have an increased risk for developing emotional and psychiatric disorders, and males have an increased risk for social and behavioral problems within the first 6–12 months following childhood TBI (88–90). In a longitudinal study of quality of life following mild-to-severe TBI sustained during childhood (91), female sex significantly predicted poorer outcomes across the majority of health-related quality of life measures as well as overall satisfaction with perceived quality of life. This conflicts with the findings above regarding hormones, suggesting that this issue may require a more nuanced approach. In particular, the impact of puberty on outcome is relatively understudied even though it plays a major role in brain development. These findings underscore the importance of considering demographic characteristics, including intersections between these variables, when assessing outcome and predicting recovery from early brain injury.

#### Socioeconomic Status

There is substantial evidence supporting the beneficial effects of an enriched environment on brain development (92–95), and more recent research has further demonstrated the effect on outcome following childhood brain injury. In a longitudinal study of functional outcome for children with TBI vs. orthopedic injury, home environment was found to moderate the effects of TBI on 7-year outcome (96). In particular, the results of this study demonstrated significantly poorer outcomes in those with TBI vs. orthopedic injury when the home environment had low enrichment (e.g., less access to educational resources and familial support), whereas both patient groups from more facilitative and enriching home environments recovered similarly well. Similar findings have been shown in other longitudinal studies, where children from higher functioning families with greater resources and more enriching home environments have better psychosocial, behavioral, and overall functional outcomes years after suffering from early brain injury (88, 97, 98). Socioeconomic status (SES) is a major determinant of how enriching one's environment is, and recent research provides evidence for the direct impact of SES on typical brain development (99, 100). Significant associations between low SES and long-term psychosocial and behavioral outcomes after pediatric TBI have also been demonstrated in the literature (101, 102).

## Longitudinal Neuroimaging of Pediatric Traumatic Brain Injury

The PubMed database was searched for English-language articles focusing on longitudinal MRI studies in young patients with a history of TBI using the following search criteria: [In Title: (pediatric OR adolescent OR child OR children OR youth)] AND [In Title: (traumatic brain injury OR brain injury OR TBI OR concussion)] AND [All Fields: (longitudinal OR chronic OR long-term OR outcome)] AND [All Fields: (MRI OR neuroimaging OR imaging)]. No time period restrictions were applied, and the latest search was undertaken on July 21, 2019. Additional searches in the references of previously published studies were conducted in an attempt to identify further articles. We excluded published study protocols, conference abstracts, articles not available in English, and experiments involving non-human subjects. The title and abstract of the retrieved articles were examined against all inclusion criteria, and the full text article was retrieved if all criteria were met. The assessment of eligibility was performed by two investigators (HML and EAW) independently with the requirement of consensus. In case of disagreement, a third expert was consulted (ELD or KC). Four longitudinal studies of sport-related concussion (SRC) in children or adolescents were excluded due to the failure to provide details or adequate definitions of concussion. In total, we identified 19 research articles that met the following four inclusion criteria: (a) the studies involved children or adolescents who sustained a TBI prior to the age of 19; (b) MRI-based methods were employed to measure brain structure and/or function; (c) changes in brain structure and/or function were assessed over at least two separate points in time (i.e., longitudinal studies). One additional study (103) was published after the initial search date and was considered for inclusion at that time, bringing the total included studies in this review to 20. Of these studies, 6 collected sMRI, 12 collected dMRI, 4 collected MRS, and 2 collected fMRI data for their longitudinal analyses.

In the following sections, we briefly describe the longitudinal studies from the current literature that evaluate change in brain structure and/or function over time using sMRI, dMRI, MRS, and/or fMRI. We will begin with a summary of the characteristics of the included studies and provide a basic description of the methods used for analysis and the outcome measures of interest. We will then summarize the overall findings of the studies for each respective imaging modality. A summary of the imaging modalities, their clinical utility in TBI populations, common outcome measures used, and the included studies that utilized them can be found in Table 1. The following data was extracted from each article, and details are summarized in Tables 2–**5**: patient and control group demographic characteristics (age and sex distribution), age and developmental stage at injury, post-injury time interval, injury severity, MR-based outcome measure(s) assessed, and analysis method(s) used. We extracted additional information regarding the racial/ethnic distribution and SES of patient and control groups, mechanism of injury, primary injuries (determined by day-of-injury CT scan), MR image acquisition details (including field strength, scanner model), and functional/behavioral domains assessed, and these details are summarized in Supplementary Tables S1–S4.

**Table 1:** Overview of the utility of magnetic resonance-based imaging modalities used in the reviewed longitudinal investigations of pediatric traumatic brain injury.

**Modality**	**Method**	**Underlying neural processes**	**Pathological sensitivity**	**Interpretation of parameters**	**Included studies**
Structural	Morphometry	Relaxation times of tissues	Regional volume	Gray matter density Jacobian determinant	(104–108)
			Cortical thickness	Thickness of cortical gray matter	(109)
	Diffusion	Brownian motion of water molecules	White matter integrity Diffuse axonal injury Demyelination Wallerian degeneration Cytotoxic edema Hemorrhage Axonal damage	**FA:** anisotropic movement of water molecules along fiber bundles; reflects white matter organization **ADC/MD:** average diffusivity; reflects the degree of overall diffusion magnitude **AD:** diffusivity along the primary axis; reflects the direction of diffusion magnitude **RD:** diffusion perpendicular to the main diffusion direction; reflects the direction of diffusion magnitude	(103, 106–108, 110–116)
			Structural connectivity	**Degree:** the number of connections between modules **MOD:** modules of connected regions that work together to support a given function **E**_**glob**_**:** how efficiently information is transferred globally throughout a network **E**_**loc**_**:** how efficiently information is transferred locally throughout a network **Betweenness centrality:** a reflection of the importance of some region for the transfer of information between other regions **Small-worldness**: a ratio reflecting the balance between network segregation and integration **Normalized clustering coefficient:** a measure of the level of segregation of the entire system	(117)
Metabolic	Spectroscopy	Intracellular metabolic status	Neuronal death Axonal damage Inflammation Demyelination Astrocytosis	**NAA:** marker of neuronal metabolism and integrity **Cho:** marker of cellular membrane turnover **Cr and Lac:** markers of energy metabolism	(110, 118–120)
Functional	Task-based	Blood oxygen level dependent (BOLD)	Functional activation	**Regional activity:** level of activity in different areas of the brain determined by BOLD signal	(121)
			Blood flow	**CVR:** change in cerebral blood flow in response to a vasodilatory stimulus	(122)

*AD, axial diffusivity; ADC, apparent diffusion coefficient; Cho, choline; Cr, creatine; CVR, cerebrovascular responsiveness; E_glob_, global efficiency; E_loc_, local efficiency; FA, fractional anisotropy; Lac, lactate; MD, mean diffusivity; MOD, modularity; NAA, N-acetyl aspartate; RD, radial diffusivity*.

**Table 2:** Summary of structural magnetic resonance imaging studies.

**Study**	**Sample characteristics**	**Age at injury**	**Time since injury**	**Severity**	**Analysis method**	**Dataset**
Dennis et al. (104)	Post-acute (T1) **TBI-Slow:** *N* = 11 (8M, 3F) Age = 14.1 ± 1.9 **TBI-Normal:** *N* = 10 (8M, 2F) Age = 16.0 ± 2.6 **HC:** *N* = 26 (15M, 11F) Age = 14.5 ± 3.0 Chronic (T2) **TBI-Slow:** *N* = 11 (8M, 3F) Age = 15.0 ± 2.0 **TBI-Normal:** *N* = 10 (8M, 2F) Age = 17.0 ± 2.8 **HC:** *N* = 26 (15M, 11F) Age = 15.6 ± 3.0	**Late Childhood—Adolescence** (ages 8–18)	T1: ~2–5 mo T2: ~14–17 mo	Moderate-Severe **TBI-Slow:** GCS = 8.8 ± 3.6 **TBI-Normal:** GCS = 9.4 ± 4.0	TBM (ANTs) **Measure:** Volume **Covariates:** age, sex, scanner, scan interval, ICV	a
Levin et al. (105)	Post-acute (T1) **mmTBI:** *N* = 28 (20M, 8F) Age = 9.7 ± 2.7 **sTBI:** *N* = 25 (15M, 10F) Age = 10.3 ± 3.1	**Early Childhood—Adolescence** (ages 5–15)	T1: ~3 mo T2: ~36 mo	Mild-Severe **mmTBI:** GCS = 13.5 ± 1.7 **sTBI:** GCS = 5.7 ± 2.0	ROI analysis (in-house software) **ROIs:** rCC, gCC, anterior bCC, middle bCC, posterior bCC, iCC, sCC **Covariates:** CC area/total brain area ratio	Ind
Mayer et al. (106)	Post-acute (T1) **TBI:** *N* = 15 (13M, 2F) Age = 13.47 ± 2.20 **HC:** *N* = 15 (12M, 3F) Age = 13.40 ± 1.84 Chronic (T2) **TBI:** *N* = 10 **HC:** *N* = 10	**Late Childhood—Adolescence** (ages 10–17)	T1: ~3 wk 15.87 ± 4.93 da T2: ~4 mo 127.82 ± 14.60 da	Mild GCS = 13–15 LOC <30 min PTA <24 h	ROI analysis (Freesurfer) **Measures:** Volume, Cortical Thickness **ROIs:** Thal, HiC **Covariates:** ICV	b
Wilde et al. (109)	Post-acute (T1) **TBI:** *N* = 20 (11M, 9F) Age = 13.6 ± 2.9 **OI:** *N* = 21 (15M, 6F) Age = 12.1 ± 2.5 Chronic (T2) **TBI:** *N* = 20 (11M, 9F) Age = 14.8 ± 2.9 **OI:** *N* = 21 (15M, 6F) Age = 13.2 ± 2.6	**Late Childhood—Adolescence** (ages 7–17)	T1: ~3 mo **TBI:** 4.0 ± 1.0 **OI:** 4.7 ± 2.6 T2: ~18 mo **TBI:** 18.5 ± 3.6 **OI:** 18.4 ± 4.2	Complicated Mild—Severe GCS = 7.9 ± 4.0 ISS = 22.6 ± 11.6	SBM (FreeSurfer) **Measure:** Cortical Thickness	c
Wu et al. (107)	Acute (T1) **SRC:** *N* = 10 (6M, 4F) Age = 14.58 ± 1.60 **OI:** *N* = 12 (9M, 3F) Age = 14.06 ± 1.70 **TDC:** *N* = 8	**Pre-adolescence—Adolescence** (ages 12–17)	T1: ~96 h 21–116 h T2: ~3 mo 84–143 da	Mild GCS = 14–15 LOC = 0–5 min PTA = 0–180 min	ROI analysis (FreeSurfer) **Measure:** Volume **ROIs:** rostral anterior CG, caudal anterior CG, posterior CG, isthmus CG, total CC **Covariates:** ICV	d
Wu et al. (108)	Post-acute (T1) **TBI:** *N* = 23 (15M, 8F) Age = 12.9 ± 3.2 **OI:** *N* = 25 (18M, 7F) Age = 11.8 ± 2.7	**Late Childhood—Adolescence** (ages 7–17)	T1: ~3 mo **TBI:** 4.0 ± 0.9 **OI:** 4.2 ± 1.0 T2: ~18 mo **TBI:** 18.9 ± 1.5 **OI:** 18.8 ± 1.3	Complicated Mild—Severe GCS = 7.5 ± 4.1	ROI analysis (Freesurfer) **Measure:** Volume **ROIs:** gCC, bCC, sCC, total CC **Covariates:** ICV	c

*Datasets that overlapped with others reviewed in this article are specified in the last column (Ind = individual study). ANTs, advanced normalization tools; bCC, body of the corpus callosum; CC, corpus callosum; F, female; CG, cingulum; gCC, genu of the corpus callosum; GCS, Glasgow Coma Scale; HC, healthy control; HiC, hippocampus; iCC, isthmus of the corpus callosum; ICV, intracranial volume; ISS, injury severity scale; LOC, loss of consciousness; M, male; mmTBI, mild/moderate traumatic brain injury; OI, orthopedic injury; PTA, posttraumatic amnesia; rCC, rostrum of the corpus callosum; ROI, region of interest; SBM, surface-based morphometry; sCC, splenium of the corpus callosum; SRC, sports-related concussion; sTBI, severe traumatic brain injury; T1, time 1; T2, time 2; TBI, traumatic brain injury; TBI-Normal, traumatic brain injury with normal inter-hemispheric transfer time; TBI-Slow, traumatic brain injury with slow inter-hemispheric transfer time; TBM, tensor-based morphometry; TDC, typically developing children; Thal, thalamus*.

### Structural Magnetic Resonance Imaging

There is a relatively liThere is a relatively limited number of longitudinal studies using structural, T_1_-weighted MRI to evaluate outcome following pediatric TBI. Six published studies were found (see Table 2), and these studies evaluated samples who were injured between early childhood and adolescence (ages 5–18). All samples were first evaluated within the subacute or acute post-injury periods, and follow-up time points occurred between 4- and 36-months post-injury. Three analysis methods were used across the six studies: four studies utilized semi- or fully automatic ROI region-of-interest (ROI) approaches to measure longitudinal changes in gray matter density (105–108), one study measured volumetric change longitudinally using tensor-based morphometry [TBM; (104)], and one study used surface-based morphometry (SBM) to measure changes in cortical thickness (109).

#### Methodology and Outcome Measurement

ROI approaches are typically used to address a specific anatomical hypothesis and involve manual or automatic segmentation of the specific region(s) to be compared between subjects. TBM (123) is an advanced whole-brain approach that involves the non-linear registration of individual subject data to a template brain space using deformation tensor fields, in which differences in the anatomical structure of each individual brain is preserved and quantified from the properties of the deformation fields via Jacobian determinants. Concerns associated with multiple comparisons exist for both whole-brain and ROI approaches (when more than one ROI is analyzed), and it is necessary to correct for this by including the appropriate statistical correction procedures. SBM is used for cortical thickness analyses and involves the initial non-linear alignment of cortical curvature across subjects and the spatial normalization data from each subject is registered to template space. Cortical thickness comparisons can then be made between subjects at homologous locations along the cortex.

#### Summary of Longitudinal Findings

**Figure 3:**
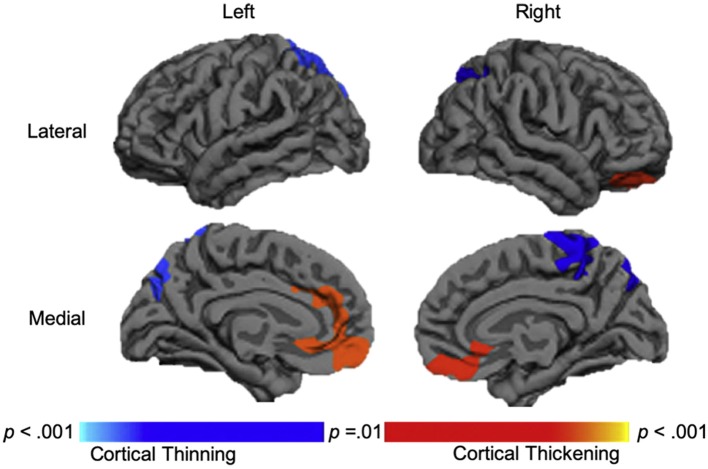
Longitudinal changes in cortical thickness in the traumatic brain injury group relative to the orthopedic injury group. Regions with relative cortical thinning are reflected by blue shading, and regions with relative cortical thickening are reflected by red-orange shading in those with traumatic brain injury over the 3-month to 18-month post-injury interval. Adapted from Wilde et al. (109).

Overall, the results of morphometric studies evaluating longitudinal change after pediatric TBI demonstrated widespread volumetric differences and cortical thinning over time, when compared to the rates of change in typically developing or orthopedically injured children of the same age. Volumetric differences, indicative of greater atrophy or cortical thinning between acute and chronic periods after pediatric TBI were consistently shown in the corpus callosum (104, 105, 108), superior and middle frontal gyri, middle temporal gyri, postcentral gyri, and lateral or middle occipital gyri (104, 106). One study, however, found no differences in morphometry between TBI and controls groups across time (107). In a supplementary analysis, in which SES was included as a covariate, Dennis et al. (104) additionally found volumetric decreases in the amygdala and middle cerebellar peduncles as well as increased volume in the superior frontal gyrus in those with TBI. In support of the findings of volumetric change over time, Wilde et al. (109) demonstrated cortical thinning in the superior parietal and right paracentral regions and cortical thickening in lateral and medial orbitofrontal regions and in the cingulate of those with TBI (see Figure 3).

**Figure 4:**
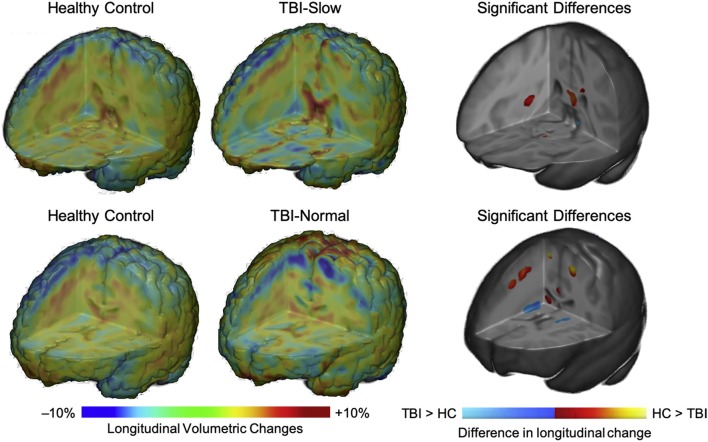
Regional volumetric changes in healthy controls vs. patients with slow interhemispheric transfer time (TBI-Slow) and healthy controls vs. patients with normal interhemispheric transfer time (TBI-Normal) groups. Longitudinal changes in regional volume are shown for healthy controls vs. TBI-Slow (top panel), and healthy controls vs. TBI-Normal (bottom panel). Colors in the group-averaged Jacobian determinants (left and center images) represent the percent of longitudinal volumetric change over a 12-month interval, according to the bottom left color bar. Beta values are overlaid on a minimal deformation template from the healthy controls, demonstrating the difference in longitudinal change, with beta values colored according to the bottom right color bar. Blue areas are those with greater increases in the TBI-Slow or TBI-Normal groups, relative to healthy controls, red-yellow areas are those with greater increases in the healthy controls, relative to the TBI groups. Images are shown in radiologic view (right = left). Adapted from Dennis et al. (104).

In a subgroup analysis, Dennis et al. (104) divided patients into TBI-Slow and TBI-Normal groups according to previously determined differences in inter-hemispheric transfer time [IHTT; see (124, 125)]. Over the first year post-injury, relative volume increases were seen in several gray matter regions in the TBI-Slow group, including the superior frontal gyrus, cingulate cortex, superior parietal lobe, parietal operculum, precuneus, cuneus, and inferior occipital gyrus. Decreased volume in several white matter regions was also seen in the TBI-Slow group, including the internal capsule (extending into the right thalamic region) and superior corona radiata. Volumetric changes in the TBI-Normal group, however, were similar to those seen in the healthy control group over the same period of time (see Figure 4). After controlling for SES, supplementary subgroup analyses revealed further volumetric decreases in the anterior corona radiata, posterior thalamic radiation, superior temporal gyrus, and precentral gyrus, and further volumetric increases in the inferior frontal and supramarginal gyri of the TBI-Slow group, relative to the healthy controls. In light of these findings, the authors suggest that trajectories of outcome might be divergent, where a subset of patients experienced relatively good recovery, characterized by developmentally-expected decreases in gray matter volume and IHTT rates that are comparable to typically developing children of the same age, whereas the other subset of patients experienced relatively poor recovery, marked by decreased white matter volume, which is reflected in slow IHTT. Although no evidence was found in support of a specific moderator for good or poor recovery trajectories, these findings clearly support a relationship between structural change and functional outcome following pediatric TBI.

### Diffusion-Weighted Magnetic Resonance Imaging

Twelve studies assessing longitudinal changes in white matter after pediatric TBI were found (see Table 3), and all of them utilized diffusion tensor imaging (DTI) to do so. All studies evaluated children who were injured between the ages of 5–18; two of these studies focused on injuries that occurred during early childhood through pre-adolescence (112, 113), while the remainder focused on children who were injured later. Apart from two studies, where children were evaluated before and after the implementation of an intervention one or more years post-injury (103, 117), all studies enrolled children during the acute or subacute phase of injury and evaluated them again between 3- and 24-months post-injury. Several analytical approaches were used to assess longitudinal change in white matter integrity across these studies: two studies used ROI analysis (106, 114), whole-brain approaches were used by four studies, including tract-based spatial statistics [TBSS; (112, 113, 116)] or fixel-based analysis [FBA; (103)], which was supplemented by ROI analysis and probabilistic tractography. The remaining seven studies used deterministic tractography (107, 108, 110, 111, 115, 117), and one of these studies (117) also implemented graph theoretical analysis to investigate longitudinal differences in structural connectivity between children with TBI and healthy controls following 10-weeks of cognitive training.

**Table 3:** Summary of diffusion-weighted imaging studies.

**Study**	**Sample Characteristics**	**Age at Injury**	**Time since Injury**	**Severity**	**Analysis Method**	**Dataset**
Dennis et al. (110)	Post-acute (T1) **TBI-Slow:** *N* = 15 (10M, 5F) Age = 13.9 ± 2.3 **TBI-Normal:** *N* = 14 (11M, 3F) Age = 13.9 ± 3.2 **HC:** N = 23 (11M, 12F) Age = 15.3 ± 2.8 Chronic (T2) **TBI-Slow:** *N* = 9 (7M, 2F) Age = 14.9 ± 2.0 **TBI-Normal:** *N* = 9 (7M, 2F) Age = 16.7 ± 2.8 **HC:** *N* = 21 (14M, 7F) Age = 15.3 ± 3.2	**Late Childhood—Adolescence** (ages 8-18)	T1: ~2-5 mo **TBI-Slow:** 12.0 ± 4.7 wk **TBI-Normal:** 12.5 ± 5.1 wk T2: ~13-19 mo **TBI-Slow:** 61.2 ± 4.8 wk **TBI-Normal:** 67.2 ± 6.1 wk	Moderate-Severe*Post-acute* **TBI-Slow:**GCS = 9.6 ± 3.9 **TBI-Normal:**GCS = 8.3 ± 4.0*Chronic* **TBI-Slow:**GCS = 7.7 ± 2.9 **TBI-Normal:**GCS = 9.4 ± 4.2	Tractography (autoMATE, Camino) **Measures:** FA, MD, AD, RD **ROIs:** CST, CGC, CGH, IFO, ILF, UF, ARC, gCC, anterior bCC, posterior bCC, iCC, sCC **Covariates:** age, sex, scanner	a
Dennis et al. (111)	Post-acute (T1) **TBI-Slow:** *N* = 11 (8M, 3F) Age = 14.1 ± 1.9 **TBI-Normal:** *N* = 10 (8M, 2F) Age = 16.0 ± 2.6 **HC:** *N* = 20 (12M, 8F) Age = 14.5 ± 3.0 Chronic (T2) **TBI-Slow:** *N* = 11 (8M, 3F) Age = 15.0 ± 2.0 **TBI-Normal:** *N* = 10 (8M, 2F) Age = 17.0 ± 2.8 **HC:** *N* = 20 (12M, 8F) Age = 15.6 ± 3.1	**Late Childhood—Adolescence** (ages 8-18)	T1: ~2-5 mo T2: ~14-17 mo	Moderate-Severe **TBI-Slow:**GCS = 8.8 ± 3.6 **TBI-Normal:**GCS = 9.4 ± 4.0	Tractography (autoMATE) **Measures:** FA, MD, AD, RD **ROIs:** ATR, CST, IFO, ILF, ARC, FX, CGC, rCC gCC, anterior bCC, posterior bCC, iCC, sCC **Covariates:** age, sex, scanner, scan interval	a
Ewing-Cobbs et al. (112)	Post-acute (T1) **TBI:** *N* = 16 (12M, 4F) **OI:** *N* = 18 (10M, 8F) Chronic (T2) **TBI:** *N* = 16 (12M, 4F) **OI:** *N* = 18 (10M, 8F)	**Late Childhood—Adolescence** (ages 6-15)	T1: ~3 mo 3.88 ± 1.63 T2: ~24 mo 24.75 ± 7.74	Mild-Severe 19% GCS = 13-15 44% GCS = 9-12 69% GCS = 3-8 ISS = 21.56 ± 10.93	TBSS (FSL) **Measures:** FA, AD, RD **ROIs:** ILF, SLF, IFO, UF, CGC, CGH, CST **Covariate:** age at injury	Ind
Genc et al. (113)	Post-acute (T1) **TBI:** *N* = 78 (50M, 28F) Age = 10.44 ± 2.21 **HC:** *N* = 30 (20M, 10F) Age = 10.60 ± 2.88 Chronic (T2) **TBI:** *N* = 15	**Early Childhood—Pre-adolescence** (ages 5-14)	T1: ~1-2 mo 5.55 ± 3.05 wk T2: ~24 mo 22.3 ± 2.3 mo	Mild-Severe GCS = 13.08 ± 2.71 LOC = 3.31 ± 3.07 hr LHS = 2.76 ± 5.33 da	TBSS (FSL) **Measures:** FA, MD, AD, RD **Covariates:** age, sex	Ind
Mayer et al. (106)	Post-acute (T1) **TBI:** *N* = 15 (13M, 2F) Age = 13.47 ± 2.20 **HC:** *N* = 15 (12M, 3F) Age = 13.40 ± 1.84 Chronic (T2) **TBI:** *N* = 10 **HC:** *N* = 10	**Late Childhood—Adolescence** (ages 10-17)	T1: ~3 wk 15.87 ± 4.93 da T2: ~4 mo 127.82 ± 14.60 da	Mild GCS = 13-15 LOC <30 min PTA <24 hr	ROI analysis (AFNI, FSL, Freesurfer) **Measures:** FA, AD, RD **ROIs:** Thal, HiC **Covariates:** ICV	b
Mayer et al. (114)	Post-acute (T1) **TBI:** *N* = 15 (13M, 2F) Age = 13.47 ± 2.20 **HC:** *N* = 15 (12M, 3F) Age = 13.40 ± 1.84 Chronic (T2) **TBI:** *N* = 11 (10M, 1F) Age = 13.82 ± 2.27 **HC:** *N* = 12 (9M, 3F) Age = 13.58 ± 1.93	**Late Childhood—Adolescence** (ages 10-17)	T1: ~ 3 wk 15.87 ± 4.93 da T2: ~4 mo 127.82 ± 14.60 da	Mild GCS = 13-15 LOC <30 min PTA <24 hr	ROI analysis (AFNI, FSL) **Measures:** FA **ROIs:** gCC, bCC, sCC, ACR, SCR, CG, IC, CP	b
Van Beek et al. (115)	Post-acute (T1) **TBI:** *N* = 20 (13M, 7F) Age = 10.8 ± 1.6 **HC:** *N* = 20 (13M, 7F) Age = 10.9 ± 1.5 Chronic (T2) **TBI:** *N* = 18 (12M, 6F) Age = 11.2 ± 1.6 **HC:** *N* = 18 (13M, 5F) Age = 11.4 ± 1.5	**Late Childhood—Pre-adolescence** (ages 7-13)	T1: ~1 mo 20 ± 7 da T2: ~6-8 mo 201 ± 22 da	Mild GCS = 13-15 LOC <30 min PTA <24 hr	Tractography (ExploreDTI, TrackVis) **Measures:** FA, MD, AD, RD **ROIs:** gCC, sCC, SLF, ILF	Ind
Verhelst et al. (103)	Pre-intervention (T1) **TBI:** *N* = 16 (9M, 7F) Age = 15.6 ± 1.8 **HC:** *N* = 16 (9M, 7F) Age = 15.6 ± 1.8 Post-intervention (T2) **TBI:** *N* = 16 (9M, 7F) **HC:** *N* = 16 (9M, 7F)	**Late Childhood—Adolescence** (ages 9-15)	T1: ≥ 12 mo 2.25 ± 1.02 T2: ~8 wk post-T1	Moderate-Severe 38% LOC ≥ 30 50% GCS <13 100% Positive CT	Tractography (MRtrix3); Whole-brain FBA (CSD); ROI analysis (TDI) **Measures:** FA, MD, FC **ROIs:** gCC, CGC, CGH, SLF-I, SLF-II, SLF-III **Covariates:** age, ICV	Ind
Wilde et al. (116)	Post-acute (T1) **TBI:** *N* = 20 (11M, 9F) Age = 13.6 ± 2.9 **OI:** *N* = 21 (15M, 6F) Age = 12.1 ± 2.5 Chronic (T2) **TBI:** *N* = 20 (11M, 9F) Age = 14.8 ± 2.9 **OI:** *N* = 21 (15M, 6F) Age = 13.2 ± 2.6	**Late Childhood—Adolescence** (ages 7-17)	T1: ~3 mo **TBI:** 4.0 ± 1.0 **OI:** 4.7 ± 2.6 T2: ~18 mo **TBI:** 18.5 ± 3.6 **OI:** 18.4 ± 4.2	Complicated Mild-Severe GCS = 7.9 ± 4.0 ISS = 22.6 ± 11.6	TBSS (FSL) **Measures:** FA, ADC	c
Wu et al. (107)	Acute (T1) **SRC:** *N* = 10 (6M, 4F) Age = 14.58 ± 1.60 **OI:** *N* = 12 (9M, 3F) Age = 14.06 ± 1.70 **HC:** *N* = 8	**Pre-adolescence—Adolescence** (ages 12-17)	T1: ~96 hr 21-116 hr T2: ~3 mo 84-143 da	Mild GCS = 14-15 LOC = 0-5 min PTA = 0-180 min	Tractography (Phillips 3D Fiber Tracking software) **Measure:** FA, ADC **ROIs:** gCC, bCC, sCC, total CC, UF, CG	d
Wu et al. (108)	Post-acute (T1) **TBI:** *N* = 23 (15M, 8F) Age = 12.9 ± 3.2 **OI:** *N* = 25 (18M, 7F) Age = 11.8 ± 2.7	**Late Childhood—Adolescence** (ages 7-17)	T1: ~3 mo **TBI:** 4.0 ± 0.9 **OI:** 4.2 ± 1.0 T2: ~18 mo **TBI:** 18.9 ± 1.5 **OI:** 18.8 ± 1.3	Complicated Mild-Severe GCS = 7.5 ± 4.1	Tractography (Philips 3D Fiber Tracking software) **Measures:** FA, ADC **ROIs:** gCC, bCC, sCC, total CC	c
Yuan et al. (117)	Pre-intervention (T1) **TBI:** *N* = 17 (10M, 7F) Age = 13.72 ± 2.77 **HC:** *N* = 11 (3M, 8F) Age = 13.37 ± 2.08 Post-intervention (T2) **TBI:** *N* = 10 **HC:** *N* = 11 (3M, 8F)	**Late Childhood—Adolescence** (ages 9-18)	T1: > 1 yr 5.91 ± 3.10 T2: ~3 mo post-T1	Complicated Mild-Severe GCS = 10.53 ± 4.8	Tractography (Diffusion Toolkit/TrackVis) + Graph Theoretical Analysis (Brain Connectivity Toolbox) **Measures:** E_glob_, E_loc_, MOD, γ, λ, σ, nodal degree, nodal clustering coefficient, nodal local efficiency, nodal betweenness centrality	Ind

*Datasets that overlapped with others reviewed in this article are specified in the last column (Ind, individual study). ACR, anterior corona radiata; AD, axial diffusivity; ADC, apparent diffusion coefficient; AFNI, Analysis of Functional Neuroimages; ARC, arcuate fasciculus; ATR, anterior thalamic radiation; bCC, body of the corpus callosum; CC, corpus callosum; CG, cingulum; CGC, cingulum cingulate; CGH, cingulum hippocampus; CP, cerebellar peduncle; CSD, constrained spherical deconvolution; CST, corticospinal tract; CT, computed tomography; E_glob_, global efficiency; E_loc_, local efficiency; F, female; FA, fractional anisotropy; FBA, fixel-based analysis; FC, fiber cross-section; FSL, FMRIB Software Library; FX, fornix; gCC, genu of the corpus callosum; GCS, Glasgow Coma Scale; HC, healthy control; HiC, hippocampus; IC, internal capsule; iCC, isthmus of the corpus callosum; ICV, intracranial volume; IFO, inferior fronto-occipital fasciculus; ILF, inferior longitudinal fasciculus; ISS, injury severity scale; LHS, length of hospital stay; LOC, loss of consciousness; M, male; MD, mean diffusivity; MOD, modularity; OI, orthopedic injury; PTA, posttraumatic amnesia; rCC, rostrum of the corpus callosum; RD, radial diffusivity; ROI, region of interest; sCC, splenium of the corpus callosum; SCR, superior corona radiata; SLF, superior longitudinal fasciculus; SRC, sports-related concussion; T1, time 1; T2, time 2; TBI, traumatic brain injury; TBI-Normal, traumatic brain injury with normal inter-hemispheric transfer time; TBI-Slow, traumatic brain injury with slow inter-hemispheric transfer time; TBSS, tract-based spatial statistics; TDC, typically developing children; TDI, track-density imaging; Thal, thalamus; UF, uncinate fasciculus; γ, normalized clustering coefficient; λ, normalized characteristic path length; σ, small-worldness*.

#### Methodology and Outcome Measurement

The most basic of the approaches used is ROI analysis, which involves the quantification of diffusion metrics within a specific area by extracting the mean parameter of interest from the voxels that fall within that region. As in volumetric analyses, ROI analyses of diffusion data are often used to address an *a priori* hypothesis but can also be used in a whole-brain approach. ROI analysis can be used to measure diffusion properties of both gray and white matter, and it can be sensitive to small changes, particularly if analyses are focused on a specific region that is prone to pathology. TBSS (126) is a whole-brain approach that involves the initial registration of subject data to template space, but this is followed by an additional step where averaged FA values from the major white matter tracts of all subjects are projected onto an alignment-invariant tract representation, called the FA skeleton. FBA is a whole-brain approach used to evaluate the organization of multiple fiber populations, or *fixels* within a single voxel (127). Fixel-based measures, such as fiber cross-section (FC), a measurement of fixel diameter, can identify tracts that are affected by regions with crossing fibers, overcoming this inherent limitation of the diffusion tensor model (128). A more recent approach toward analyzing white matter microstructure is tractography, which is used to reconstruct individual white matter pathways from tensor field data embedded within the underlying voxels. Measures of anisotropy and diffusivity are be sampled from various regions or across the entire reconstructed tract. Tractography has advantages over the other approaches described, majorly due to the fact that it does not necessarily rely on the registration of subject data to template space. Rather, subject data can be analyzed individually, and this allows for the assessment of interindividual differences in white matter pathology that cannot be obtained through whole-brain, voxel-wise approaches toward diffusion data analysis. Finally, tractography can also be used in combination with cortical parcellation maps obtained through morphometric analyses to create structural connectivity maps using graph theory (129). Whole brain structural connectivity can be modeled as a complex structural network and depicted as graphs composed of nodes and edges, where the nodes represent anatomical regions or voxels, and the edges are reflected in white matter fiber bundles representing the structural connectivity between nodes. Graph theory allows for the analysis of complex networks in which multimodal neuroimaging can be used to characterize the topological properties of brain connectivity through commonly used measures of local and global network connectivity, which are described in Table 1 [for a review, see (130)].

Common diffusion metrics used in tensor-based approaches (i.e., ROI analysis, TBSS, tractography) include fractional anisotropy (FA), apparent diffusion coefficient (ADC, also called mean diffusivity–MD), and axial and radial diffusivity (AD and RD, respectively). FA is highly sensitive to the presence of disorganized white matter; however, it cannot identify specific changes in shape or distribution of the diffusion tensor ellipsoid and should therefore not be considered a biomarker of white matter integrity when interpreted alone (131). ADC/MD reflect the degree of overall diffusion magnitude, and changes in ADC/MD reflect variations in the ratio of intra- to extracellular water concentrations, whereas AD and RD more precisely describe the directional magnitude of diffusion. Variations in ADC/MD in white matter are suggestive of changes in fiber density, axonal diameter, myelination, and neuronal or glial loss, whereas decreased ADC/MD in the gray matter has been attributed to cytotoxic edema. Increases in FA that result from decreases in both AD and RD are suggestive of axonal degeneration (132, 133), whereas decreases in FA resulting from increased RD without change in AD suggests demyelination or Wallerian degeneration (134, 135). When considering brain maturation or recovery, increases in AD accompanied by decreases in RD are suggestive of axonal restoration that is preceded by remyelination, and such processes are often shown to occur with a gradual increase in FA (136, 137), though the specificity of these metrics, and their relation to specific forms of pathology requires additional investigation.

#### Summary of Longitudinal Findings

Overall, mixed results were seen in terms of longitudinal changes in white matter integrity following pediatric TBI. Mayer et al. (106) conducted a vertex-wise ROI analysis to investigate microstructural changes in gray matter regions. Despite long-term changes in gray matter density (see the summary of sMRI findings), no changes in FA from 3-weeks to 4-months post-injury were seen in the thalamus or hippocampi of the mTBI group, relative to the healthy controls. The authors suggest that these results, albeit inconclusive, might indicate differential time-courses of recovery for FA and gray matter density following pediatric mTBI. In an earlier study conducted on the same sample, Mayer et al. (114) used ROI analysis to investigate diffusion abnormalities in white matter following pediatric mTBI and found significant FA increases between 3-weeks to 4-months post-injury for the mTBI patients, relative to healthy controls, in the genu, body, and splenium of the corpus callosum, the right anterior thalamic radiation, and bilaterally in the superior corona radiata, internal capsules, cingulum bundles, and cerebral peduncles.

Ewing-Cobbs et al. (112) used TBSS to evaluate change in FA, AD, and RD between 3- and 24-months post-injury in younger (~8 years), middle (~10 years), or older (~13.5 years) children at the time of injury. In the sample of children who completed the scans at both time points, a significant increase in FA was seen over time in the left corticospinal tract, where FA was consistently lower in children who sustained a TBI at a younger age, but FA increased at a lesser rate over time in the children who sustained a TBI at an older age. These results suggest that children who are injured at an earlier age recover more quickly than those injured at later ages. Genc et al. (113) used TBSS to address the impact of injury severity on changes in FA and diffusivity over the first 2 years post-injury. Their results demonstrate that injury severity predicts increases in MD of the genu of the corpus callosum, right superior longitudinal fasciculus, retrolenticular internal capsule, and anterior and posterior corona radiata over time. Injury severity also predicted increased AD in the genu of the corpus callosum and left anterior corona radiata, as well as increased RD in the right posterior corona radiata, although no associated were seen between injury severity and changes in FA of any pathway. In contrast to the results of Ewing-Cobbs et al. (112), longitudinal changes in diffusivity were not moderated by age at injury; however, a positive relationship between age at evaluation and rate of increase in MD, AD, and RD over time was seen. Wilde et al. (116) used TBSS to evaluate changes in FA and ADC of white matter and subcortical structures in pediatric msTBI vs. patients with orthopedic injury over a period of 3- to 18-months post-injury, and their results suggest different rates of change over time across several structures. In the msTBI group, decreased FA occurred along with increased ADC in the anterior temporal white matter, genu of the corpus callosum, and parietal white matter, which suggests continued degeneration in these regions. Decreases in both FA and ADC were seen over time in the frontal and parietal white matter, splenium of the corpus callosum, brainstem, and cerebellum of those with msTBI, which may be attributed to ongoing changes that result from secondary brain injury mechanisms. These findings are compared to those seen in the orthopedic injury group over the same period of time, where general increases in FA and decreases in ADC were seen across the majority of regions, presumably reflecting developmental myelination, which is typical of healthy individuals within this age group.

**Figure 5:**
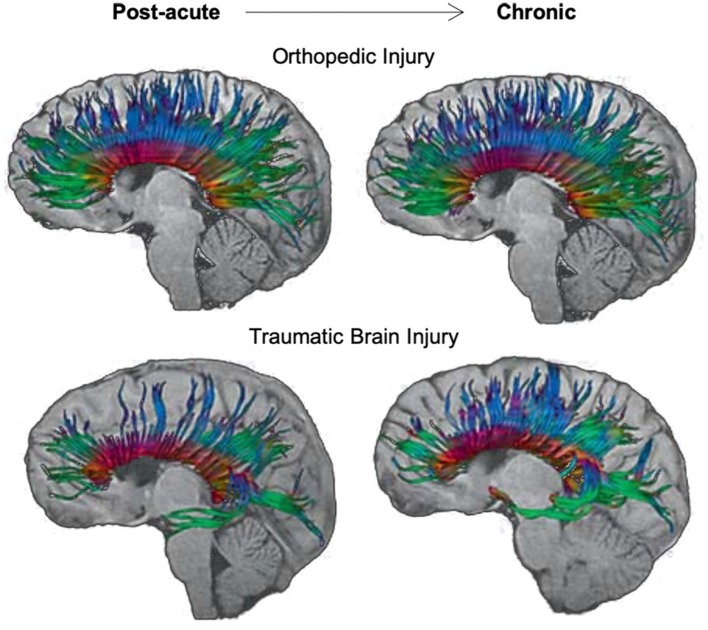
Longitudinal changes in diffusion tensor imaging (DTI) tractography of the corpus callosum in an adolescent with orthopedic injury (OI) vs. an adolescent with moderate traumatic brain injury (TBI). DTI tractography is shown at post-acute (~3 months post-injury) and chronic (~18 months post-injury) periods. The top panel of images reflects the corpus callosum in a 16-year-old male with OI, and the bottom panel of images reflects the corpus callosum in a 15-year-old male with moderate TBI (GCS = 9). Note the subtle atrophy amidst development of the corpus callosum in the adolescent with moderate TBI over time. Adapted from Wu et al. (108).

Using tractography, Van Beek et al. (115) found decreased FA and increased RD in the genu and splenium of the corpus callosum of children with mTBI, relative to controls, over the first 8 months post-injury. These changes occurred along with relatively poorer verbal working memory abilities in the mTBI group, which suggests a deficit in the development of these skills over time, possibly due to the slow maturation of commissural white matter fibers relative to healthy children of the same age. Similar results were seen in a study by Wu et al. (107), where decreases in FA and increases in ADC were seen over the first 3-months post-injury in the splenium and total corpus callosum of adolescents with sports-related concussion compared to those with orthopedic injury and healthy adolescents. A similar examination in a group of children or adolescents with complicated mild-to-severe TBI by Wu et al. (108) revealed increases in ADC of the splenium of the corpus callosum in those with TBI over a period between 3- and 18-months post-injury; however, FA increased at a similar rate across both TBI and orthopedic injury groups during this same period. These findings suggest that while similar rates of maturation occurred over time in the corpus callosum for all participants, some level of atrophy also occurred in this region for those with pediatric TBI relative to controls (see Figure 5). Although no significant differences were observed in processing speed abilities of these two groups, a negative relationship between increased ADC in the splenium and decreased processing speed abilities was evident in the TBI group.

**Figure 6:**
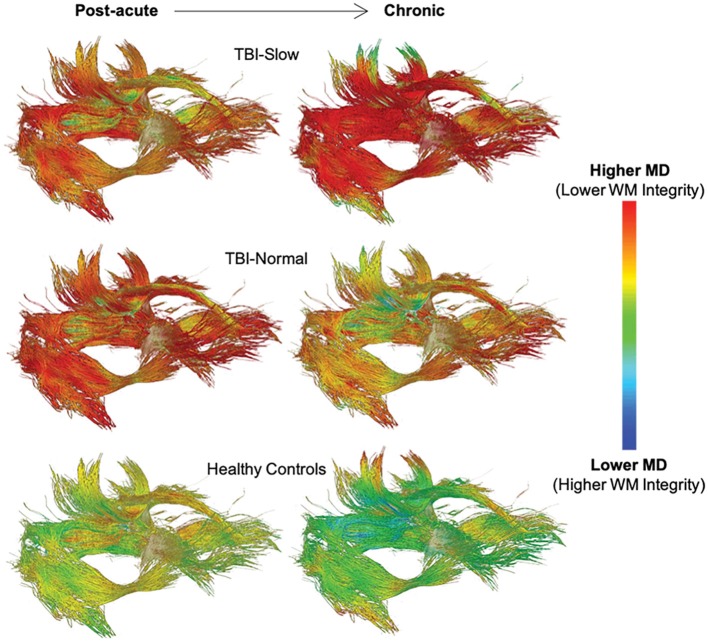
Longitudinal changes in mean diffusivity (MD) along white matter tracts in traumatic brain injury (TBI) patients with slow interhemispheric transfer time (TBI-Slow), TBI patients with normal interhemispheric transfer time (TBI-Normal), and healthy controls. Tract-averaged maps are shown for each group at the post-acute (~2–5 months post-injury) and chronic (~13–19 months post-injury) periods, during which time ~12 months passed. As indicated in the legend, areas with the lowest MD, and therefore the highest white matter integrity, are blue, whereas areas with the highest MD, and therefore the lowest white matter integrity, are red. The healthy controls (*n* = 20) show minimal decreases in MD, while widespread increases in MD are seen in the TBI-Slow group (*n* = 11), and a mixture of these patterns are seen in the TBI-Normal group (*n* = 10). Adapted from Dennis et al. (111).

The relationship between white matter integrity and processing speed over time is supported by the results of sub-group analyses based on IHTT differences in adolescents with TBI [see (124, 125)]. For example, Dennis et al. (111) found a decline in white matter integrity, marked by increased MD, RD, and AD, in the anterior midbody, posterior midbody, isthmus, and splenium of the corpus callosum, fornix, left cingulum, left arcuate, and bilateral anterior thalamic radiations, inferior fronto-occipital fasciculi, and inferior longitudinal fasciculi in the TBI-Slow group over the first year post-injury; these changes were not seen in the TBI-Normal or healthy control groups (see Figure 6). In a different experiment using a subset of the same sample, Dennis et al. (110) used DTI tractography along with MRS to demonstrate that decreases in FA, due to increases in MD and RD, were only present in tracts of the TBI-Slow group. The TBI-Slow group also showed longitudinal abnormalities in metabolic levels of *N*-acetyl aspartate, which is indicative of poorer neuronal health (as discussed in the next section).

Two studies used dMRI to evaluate changes in microstructural integrity in pediatric TBI that occur following a cognitive intervention. Verhelst et al. (103) used a whole-brain FBA approach to assess the effects of restorative cognitive training on white matter integrity in children and adolescents who sustained a TBI at least 12 months prior. Their results indicated no significant differences in FA, MD, or FC in any white matter tracts of interest in the patient group following 8-weeks of participation in an intervention designed to improve attention, working memory, and executive function. In terms of the relationship between structural and functional change following training, however, improvement on a task of verbal working memory was significantly associated with reduced MD in the left superior longitudinal fasciculus, and improvement on a task of visual processing speed was significantly associated with increased FA in a cluster of fixels in the right precentral gyrus. Based on their overall findings, the authors suggest that functional recovery may precede structural recovery, and longer periods of cognitive training may be necessary for underlying structural changes to occur. The results of a similar investigation of network changes in structural connectivity following 10-weeks of attention and executive function training do not fully support this idea, however. Using graph theoretical analysis, Yuan et al. (117) found that initially elevated small-worldness and normalized clustering coefficient were significantly reduced following training in children with TBI, such that small-worldness more closely approximated that of the healthy controls, and these structural changes occurred along with improved performance on measures of attention and executive function. Considering the training-induced reductions in normalized clustering coefficient that were also seen in the TBI group, the authors argue that the network response to the intervention was likely driven by small, local (rather than long-distance) changes in structural connectivity that occurred throughout the network. The resulting reduction in small-worldness suggests that a partial normalization of the balance between segregation and integration throughout the structural network, which is crucial for efficient communication between brain regions, may be triggered by cognitive training several years after pediatric TBI. While the results of these ample intervention studies are not consistent in terms of the extent to which structural changes may occur following a short-term intervention, important clinical implications for the effectiveness of cognitive rehabilitation long after pediatric TBI are nonetheless demonstrated. The results of these studies shed light on the potential benefit of restorative cognitive training for improving long-term outcome and recovery. However, future research is required to determine whether such effects reliably extend beyond functional restoration and contribute to the reorganization of underlying brain structure following pediatric TBI.

### Magnetic Resonance Spectroscopy

Four longitudinal studies (see Table 4) used MRS to evaluate changes in metabolic levels following pediatric TBI. Participants were enrolled during the post-acute phase following injury and follow-up visits took place at 4-, 12-, or 18-months after TBIs sustained during childhood or adolescence (age at injury ranging from 4 to 18 years). Several analysis methods exist for MRS, however only two are used in the studies covered in the present review. Two of the four studies (119, 120) implemented multivariate (MV) single slice approaches through an automated spectra quantification method, whereas the other two studies utilized a whole-brain approach (110, 118).

**Table 4:** Summary of magnetic resonance spectroscopy studies.

**Study**	**Sample characteristics**	**Age at injury**	**Time since injury**	**Severity**	**Analysis method**	**Dataset**
Babikian et al. (118)	Post-acute (T1) **TBI-Slow:** *N* = 14 (9M, 5F) Age = 14.0 ± 2.5 **TBI-Normal:** *N* = 17 (13M, 4F) Age = 14.3 ± 3.3 **HC:** *N* = 48 (23M, 25F) Age = 15.6 ± 2.7 Chronic (T2) **TBI-Slow:** *N* = 12 **TBI-Normal:** *N* = 12 **HC:** *N* = 35	**Late Childhood—Adolescence** (ages 8–18)	T1: ~3 mo **TBI-Slow:** 11.6 ± 4.2 wk **TBI-Normal:** 12.6 ± 5.5 wk T2: ~16 mo **TBI-Slow:** 63.7 ± 7.8 wk **TBI-Normal:** 69.5 ± 8.0 wk	Moderate-Severe **TBI-Slow:**Initial GCS = 8.9 ± 3.6Low GCS = 7.0 ± 3.6 **TBI-Normal:** Initial GCS = 8.2 ± 3.9Low GCS = 8.3 ± 4.0	Whole-brain (MIDAS) **Measures:** NAA, Cho, Cr **ROIs:** Frontal, Temporal, Parietal, and Occipital GM, gCC, bCC, sCC, HiC, CB	a
Dennis et al. (110)	Post-acute (T1) **TBI-Slow:** *N* = 15 (10M, 5F) Age = 13.9 ± 2.3 **TBI-Normal:** *N* = 14 (11M, 3F) Age = 13.9 ± 3.2 **HC:** *N* = 23 (11M, 12F) Age = 15.3 ± 2.8 Chronic (T2) **TBI-Slow:** *N* = 9 (7M, 2F) Age = 14.9 ± 2.0 **TBI-Normal:** *N* = 9 (7M, 2F) Age = 16.7 ± 2.8 **HC:** *N* = 21 (14M, 7F) Age = 15.3 ± 3.2	**Late Childhood—Adolescence** (ages 8–18)	T1: ~2–5 mo **TBI-Slow:** 12.0 ± 4.7 wk **TBI-Normal:** 12.5 ± 5.1 wk T2: ~13–19 mo **TBI-Slow:** 61.2 ± 4.8 wk **TBI-Normal:** 67.2 ± 6.1 wk	Moderate-Severe *Post-acute* **TBI-Slow:**GCS = 9.6 ± 3.9 **TBI-Normal:**GCS = 8.3 ± 4.0*Chronic* **TBI-Slow:**GCS = 7.7 ± 2.9 **TBI-Normal:**GCS = 9.4 ± 4.2	Whole-brain (MIDAS, autoMATE) **Measures:** NAA, Cho **ROIs:** CST, CGC, IFO, ILF, UF, CGH, ARC, gCC, anterior bCC, posterior bCC, iCC, sCC **Covariates:** age, sex, scanner	a
Holshouser et al. (119)	Post-acute (T1) **mmTBI:** *N* = 32 (26M, 6F) Age = 12.2 ± 3.3 **sTBI:** *N* = 32 (21M, 11F) Age = 12.0 ± 3.8 **HC:** *N* = 63 (33M, 30F) Age = 12.6 ± 3.3	**Early Childhood—Adolescence** (ages 4–18)	T1: ~2 wk **mmTBI:** 11.7 ± 3.2 da **sTBI:** 11.5 ± 3.7 da T2: ~12 mo **mmTBI:** 12.2 ± 0.96 mo **sTBI:** 12.1 ± 0.63	Complicated Mild—Severe**mm** **TBI:**GCS = 13.6 ± 2.0LOC = 0.63 ± 0.55da LHS = 5.88 ± 3.01 da**s** **TBI:**GCS = 4.4 ± 1.8LOC = 5.53 ± 5.81da LHS = 17.4 ± 10.1 da	MV single slice (LCModel) **Measures:** NAA, Cho, Cr, Lac **ROIs:** Frontal, temporal, parietal, and occipital GM and WM, BG, BS, CC, Thal, CB **Covariate:** age	Ind
Yeo et al. (120)	Initial (T1) **TBI:** *N* = 36 (30M, 6F) Age = 13.62 ± 3.59 **HC:** *N* = 14 (4M, 10F) Age = 15.29 ± 1.60 Follow-up (T2) **Short TI:** *N* = 13 **Medium TI:** *N* = 14 **Long TI**: *N* = 9	**Late Childhood—Adolescence** (ages 6–18)	T1: ~3.5 wk 35.46 ± 33.95 da T2: ~5, 13, or 24 wk **Short TI:** 1.50 ± 2.46 wk **Medium TI:** 9.29 ± 2.73 wk **Long TI:** 21.22 ± 6.22 wk	Complicated Mild-SevereGCS = 8.11 ± 4.60	MV single slice (LCModel) **Measures:** NAA, Cho, Cr **ROIs:** Anterior and posterior compartments of supraventricular slab	Ind

*Datasets that overlapped with others reviewed in this article are specified in the last column (Ind, individual study). ARC = arcuate fasciculus; bCC, body of the corpus callosum; BG, basal ganglia, BS, brainstem; BS, brainstem; CB, cerebellum; CC, corpus callosum; CGC, cingulum bundle cingulate; CGH, cingulum bundle hippocampal; Cho, choline; Cr, creatine; CST, corticospinal tract; F, female; gCC, genu of the corpus callosum; GCS, Glasgow Coma Scale; GM, gray matter; HC, healthy control; HiC, hippocampus; iCC, isthmus of the corpus callosum; IFO, inferior fronto-occipital fasciculus; ILF, inferior longitudinal fasciculus; Lac, lactate; LHS, length of hospital stay; LOC, loss of consciousness; M, male; MIDAS, Metabolite Imaging and Data Analysis System; mmTBI, mild/moderate traumatic brain injury; MV, multi-voxel; NAA, N-acetyl aspartate; sCC, splenium of the corpus callosum; sTBI, severe traumatic brain injury; T1, time 1; T2, time 2; TBI, traumatic brain injury; TBI-Normal, traumatic brain injury with normal inter-hemispheric transfer time; TBI-Slow, traumatic brain injury with slow inter-hemispheric transfer time; Thal, thalamus; TI, time interval; UF, uncinate fasciculus; WM, white matter*.

#### Methodology and Outcome Measurement

MV single slice approaches involve the simultaneous acquisition of multiple spectroscopic voxels arranged in a grid across a predetermined volume of interest (VOI), from which the spectrum of metabolites can be mapped. The MV single slice approach has the advantage of simultaneous assessment of multiple tissues or multiple lesions present in a specified VOI. Furthermore, this approach is capable of showing changes in the composition of metabolites across the included voxels, which allows for good predictive value in determining the margins surrounding a lesion. Due to difficulties that arise in the shimming procedure that is required for the acquisition of a robust metabolic spectrum, the precision of voxels is degraded, and partial volume errors commonly occur. Such disadvantages have led researchers to use other approaches toward analyzing MRS data, and whole-brain approaches have recently been implemented as an alternative. Whole-brain approaches use similar processes as those used in TBM for structural MR data. The Metabolite Imaging and Data Analysis System [MIDAS; (138)] pipeline allows users to generate robust, spectrally fit data from Fourier transform reconstruction and automated spectral fitting. A water-reference MRS dataset is then used to calibrate the spectrally fit data before it is normalized and registered to a common template space. This procedure is capable of maintaining the accuracy of the acquired neurochemical concentrations across the entire brain and includes a quality assurance check to correct for CSF partial-volume signal loss, which gives the whole-brain approach an advantage over MV single slice methods.

Key metabolites measured by MRS include *N*-acetyl aspartate (NAA), choline (Cho), creatine (Cr), and lactate. NAA is an amino acid produced by neuronal mitochondria that is believed to be an indicator of neuronal metabolism and integrity. In the developing brain, NAA is involved in myelin synthesis (139). In adults, NAA is involved in axonal repair, thus it is a good marker of axonal or neuronal integrity (140). Decreases in NAA are generally suggestive of neuronal death (141) and have been used as an indicator of disrupted myelin in damaged, developing brains (142). Cho levels are elevated postnatally, but decrease rapidly as the brain matures, and increased levels after birth are suggestive of inflammation, demyelination, or membrane synthesis/repair (140, 142). Both Cr and lactate are markers of energy metabolism. Imbalances in Cr concentration have been seen in mTBI (143, 144) and msTBI (145), although the directional nature is inconsistent in the literature. Furthermore, the causality of this imbalance has not been determined, though it has been suggested that changes in Cr concentrations are related to maintaining various equilibriums in the brain (146). Elevations in lactate, however, have been shown to indicate tissue damage due to ischemia, hypoxia, or inflammation (147). Increased Cho in white matter can result from cellular breakdown from shearing injuries or astrocytosis, suggesting DAI, and decreased NAA is typically the result of axonal damage. Further, an increased ratio of Cho/Cr commonly accompanies subarachnoid hemorrhage (148) and is related to poor long-term outcome after pediatric msTBI (149, 150).

#### Summary of Longitudinal Findings

Overall, the results of the four MRS studies reviewed here consistently demonstrate subacute decreases in NAA or NAA/Cr with simultaneous increases in Cho or Cho/Cr across white matter, gray matter, and subcortical regions, which likely reflects the primary injury-induced metabolic cascade that reduces the integrity of affected neurons and axons, leading to inflammation or alterations in membrane metabolism. Likewise, studies consistently demonstrate that the initial metabolic changes generally return to normal levels during the chronic phase of recovery (between 6 and 12 months). Yeo et al. (120) evaluated recovery at 5-, 13-, and 24-weeks post-injury and found that this trajectory of metabolic recovery was only present in the subset of patients who followed up at 24-weeks, and no significant changes had yet occurred in those who followed-up at earlier time points. Similar results were reported by Holshouser et al. (119), where acutely altered metabolic levels returned to normal after 1 year across all gray and white matter regions in patients who had sustained early complicated mTBI or moderate TBI. In the severe TBI group, however, metabolic levels only returned to normal in cortical gray matter regions, whereas NAA/Cr and NAA/Cho ratios remained significantly lower in hemispheric white matter and, to a somewhat lesser extent, in subcortical regions. The authors suggest that these findings may be a reflection of neuroinflammation or an indication of recovery with cellular proliferation. Further investigation revealed that, when considered together, acute subcortical NAA/Cr ratios and length of hospital stay are accurate predictors of long-term neurological and neuropsychological recovery from early TBI (*R*^2^ = 47.6) and can be used for the successful classification of TBI with 71.4% sensitivity and 96% specificity.

In addition to supporting the overall recovery of metabolic activity over the first year using a whole-brain approach, Babikian et al. (118) further employed a subgroup analysis in their TBI sample based on IHTT differences [see (124, 125)]. While the TBI-Normal group's metabolic levels of Cho returned to normal levels chronically, NAA levels in the corpus callosum were increased above those of the healthy control group, supporting a relationship between the recovery of metabolic activity in the commissural white matter and faster IHTT. In contrast, metabolic levels in the TBI-Slow group, who suffer from significantly slower IHTT, did not recover over a period of 3- to 18-month post-injury. Rather, the TBI-Slow group was shown to have lower levels of Cho globally and lower levels of NAA in the corpus callosum, relative to the TBI-Normal group. These findings suggest that the acute metabolic abnormalities, reflective of initial neuronal loss and impaired oligodendrocyte/myelin function, do not recover over time in those with functional impairments evidenced by slower IHTT; furthermore, the lower levels of Cho longitudinally in this group suggest a lack of ongoing membrane repair. These results are extended by Dennis et al. (110), who used multimodal MRI imaging to investigate the relationship between long-term metabolic differences in relation to white matter microstructure between the same IHTT subgroups of this pediatric TBI sample. Using MRS in combination with DTI tractography, the authors replicated the previous findings of Babikian et al. (118), but extended them by demonstrating that the specific white matter pathways with lower NAA in the TBI-Slow group also showed lower FA resulting from higher MD and/or RD, which is indicative of demyelination (134, 135). Such findings highlight the utility of multi-modal investigations of recovery from early TBI.

### Functional Magnetic Resonance Imaging

Currently, only two longitudinal fMRI studies have been published in the pediatric TBI literature (see Table 5). Both studies investigated adolescents who were injured around four months prior to the initial visit, and follow-up visits occurred around 8- or 16-months post-injury. Cazalis et al. (121) implemented a spatial working memory paradigm in their task-based fMRI analysis of 6 adolescents with complicated mild-to-severe injuries, whereas Mutch et al. (122) used CO_2_ stress testing and fMRI to assess whole-brain CVR in 6 adolescents with mild SRC relative to 24 healthy individuals between the ages of 13 and 25.

**Table 5:** Summary of functional magnetic resonance imaging studies.

**Study**	**Sample Characteristics**	**Age at Injury**	**Time since Injury**	**Severity**	**Analysis Method**	**Dataset**
Cazalis et al. (121)	Post-acute (T1) **TBI:** *N* = 6 (5M, 1F) Age = 16.7 ± 0.7 Chronic (T2) **TBI:** *N* = 6 (5M, 1F) Age = 17.7 ± 0.8	**Adolescence** (ages 15-17)	T1: 3–6 mo 4.5 ± 1.0 mo T2: ~15–18 mo 16.5 ± 3.1 mo	Complicated Mild-Severe GCS = 9.4 ± 5.1	Task-based ROI analysis (FSL) **Measure:** Working Memory Load (response accuracy + reaction time across four conditions) **ROIs:** Left SMC (MNI *xyz* = −40, −32, 56; 20 mm radius), ACC (MNI *xyz* = 0, 34, 28; 14 mm radius)	a
Mutch et al. (122)	Initial (T1) **SRC:** *N* = 6 (3M, 3F) Age = 15.67 ± 0.82 **HC:** *N* = 24 (15M, 9 F) Mean age = 18.5	**Adolescence** (ages 15-17)	T1: 115.7 ± 113.6 da T2: 218.8 ± 150.3 da	Mild ICCS guidelines	Whole-brain CVR mapping (SPM) **Measures:** CVR	Ind

*Datasets that overlapped with others reviewed in this article are specified in the last column (Ind, individual study). ACC, anterior cingulate cortex; CVR, cerebrovascular responsiveness; F, female; FSL, FMRIB Software Library; GCS, Glasgow Coma Scale; HC, healthy control; ICCS, International Consensus on Concussion in Sports; M, male; MNI, Montreal Neurological Institute; ROI, region-of-interest; SMC, sensorimotor cortex; SPM, Statistical Parametric Mapping; SRC, sports-related concussion; T1, time 1; T2, time 2; TBI, traumatic brain injury*.

#### Methodology and Outcome Measurement

Functional MRI measures signal variations in the blood-oxygen-level-dependent (BOLD) hemodynamic response, which indicates active regions of the brain during task-based fMRI paradigms. Basic task-based fMRI designs include block and event-related designs. Block designs involve the constant presentation of some stimulus or task during a specific block of time, followed by a period of rest; this pattern is repeated several times in an alternating fashion. Event-related designs are similar, but the stimulus or task occurs at random intervals and varies in the duration of presentation time. While block designs are more powerful, event-related designs are more flexible and more sensitive to the shape of the hemodynamic response; for this reason, event-related designs are more commonly used in the present literature. Impairments in the system involved in the control and regulation of cerebral blood flow have been noted in TBI (151), and any change in cerebral blood flow in response to a vasodilatory stimulus, or cerebrovascular responsiveness (CVR), can be used to measure the functional status of this system (152, 153). Recent work by Mutch et al. (154) has led to the development of MR-based CO_2_ stress testing, in which CO_2_, a quantifiable and reliable vasoactive stimulus (155), is administered during BOLD fMRI, allowing for the standardized measurement of CVR longitudinally.

#### Summary of Longitudinal Findings

In line with the results of the majority of the studies using other imaging modalities that have been covered in this review, studies using fMRI have found general patterns of normalization in brain function over the course of time following pediatric TBI, although several factors appear to be involved in the degree and extent to which recovery occurs. Using task-based fMRI, Cazalis et al. (121) found that as patients with complicated mild-to-severe TBI progressed into the chronic phase of recovery, a partial normalization of acutely increased anterior cingulate cortex activity occurred along with a simultaneous increase in left sensorimotor cortex activity during participation in a difficult working memory task, which better represented the activations patterns seen in the healthy adolescents at the initial visit. These longitudinal changes in brain activity in the patients with TBI were accompanied by improvements in processing speed, although no improvements were seen in working memory ability. It is important to note that covarying for task performance is recommended if it differs between groups. Following an in-depth discussion of conflicting models in the literature for the role of the anterior cingulate cortex after pediatric msTBI, the authors suggest that, based on the results of their study, the anterior cingulate cortex may play a compensatory role in recovery from pediatric TBI, where it is recruited when the executive system is overloaded during participation in a difficult task or when structural disconnection has occurred.

In their longitudinal investigation of whole-brain CVR following SRC, Mutch et al. (122) found predominantly increased patterns of CVR in the subacute phase; however, during the chronic phase, significantly decreased levels of CVR were seen in all adolescents with SRC, relative to healthy individuals. Interestingly, a stable pattern of decreased CVR was seen in two patients with chronic vestibulo-ocular and psychiatric symptoms, whereas slight improvements, that nevertheless remained persistently abnormal relative to healthy individuals, were seen in the remaining four patients, who either fully recovered or demonstrated relatively mild post-concussive symptomology. The findings of this pilot study highlight the potential utility of CVR as a marker of long-term recovery from SRC, in which the stability of CVR patterns during the chronic phase may be indicative of the degree of recovery that has occurred.

### Methodological Considerations

A major challenge in studying the changes in brain structure following injury is characterizing how damage to pathway microstructure evolves over time and interacts with ongoing developmental changes. Unmyelinated axons are highly vulnerable to injury, and the rapid, ongoing myelination of most pathways may confer particular vulnerability when injury is sustained during the early stages of development (112). Age at the time of the injury and the amount of time that has elapsed post-injury interact, complicating the changing trajectories of anisotropy and diffusion. The trajectory of change over time must be compared to what is expected at different developmental stages, thus longitudinal studies are necessary to emphasize the dynamic and disruptive interplay of early brain injury and the subsequent development of neuronal processes, such as axonal thinning and increased myelination (19). Due to the initial increase and subsequent decrease in gray matter volume and the steady increase in white matter maturation that occurs during typical brain development across childhood and adolescence, the interpretation of longitudinal structural and functional changes after pediatric TBI is inherently more complex than that of recovery from adult TBI (156, 157). Care must be taken to ensure that appropriate factors, such as age at injury, age at enrollment, sex, time-since injury, and scan interval are considered in the longitudinal analysis of structural brain changes; differences in intracranial volume (ICV) are also necessary to control for when assessing morphometric changes. While all sMRI studies included ICV as a covariate, none of the 20 studies presently reviewed controlled for the effects of time-since-injury in their analyses. The effects of age at the time of injury were controlled for in one study (112), the effects of age at the time of enrollment were controlled for in six studies (103, 104, 110, 111, 113, 119), the effects of sex were controlled for in four studies (104, 110, 111, 113), and the effects of scan interval were controlled for in two studies (104, 111).

In addition to the necessity of controlling for the factors specified above, it is necessary that data is collected regarding other factors known to influence recovery and quantitative neuroimaging, *per se*; in particular, detailed documentation of SES, injury severity classification, mechanism of injury, and lesion characteristics for primary injuries found on initial neuroimaging should be obtained and reported when publishing pediatric TBI research. Although racial or ethnic background was not discussed as a factor influencing outcome, epidemiological studies suggest that disparities exist in the prevalence, severity, and mechanism of injuries sustained by children from different racial or ethnic groups. According to these studies, African American, Hispanic, and Native American children are more likely to be hit by motorized vehicle as a pedestrian or cyclist, experience msTBI, and have higher rates of mortality than Caucasian children, regardless of SES (158–160). For reasons such as these, it is important to report the racial or ethnic distribution of pediatric TBI samples in research (refer to the Supplementary Material for details regarding the reporting of such information in the included studies). While eleven studies included a measure of SES [e.g., parental education or SES composite indices; (104–109, 112–114, 116, 118)], two of these studies did not include SES results in their sample description (104, 107); however both studies reported no differences between SES of their TBI and control samples. The distribution of various injury mechanisms were reported for the pediatric TBI sample and orthopedic injury samples in all but four studies (117, 118, 120, 121), and specific information regarding the abnormal results found on day-of-injury neuroimaging was reported by all but eight studies (107–109, 115, 117–119). Additionally, one study reported complications seen on susceptibility weighted-imaging obtained at the initial evaluation, which occurred at least 12-months post-injury (103). While all studies provided the criteria used to classify injury severity, six studies did not comprehensively report the results of the measures (i.e., descriptive statistics) used to determine the injury severity in their pediatric TBI or SRC samples (103, 106, 107, 114, 115, 122). Finally, of the twenty longitudinal studies presently reviewed, only five provided information regarding the racial or ethnic distribution of their samples (108, 109, 112, 116, 117).

Several other methodological considerations must be addressed among the studies included the present review. In a field that often publishes findings from studies with small sample size, it is important that sample characteristics are reported in adequate detail so that meta-analytic studies can be performed, and meaningful results can be derived from the published data. Detailed descriptive statistics for all demographic characteristics across all samples, and for the injury characteristics of the injured samples, must be provided; this is especially true of longitudinal studies in children, in which attrition often leads to non-random differences in sample characteristics between initial and follow-up evaluations and samples continue to develop over time (known as “attrition bias”). For example, SES (including both educational and occupational attainment, level of income, and social class) has been cited as a contributing factor for continued participation in long-term studies generally (161, 162) and in studies of pediatric TBI in particular (163). In studies of children, complex health, motivational, and lifestyle factors for both parent and child may also affect continued participation, and these factors may or may not change over long follow-up periods (162). Estimates of attrition are variably reported, but non-imaging studies of pediatric TBI have reported attrition estimates that range from 20% to over 60% (164–166).

Small sample sizes, the failure to report sufficient descriptive statistics, and attrition rates are sources of potential bias that may threaten internal and external validity, and necessary steps to avoid them must be undertaken during study design and data collection in future longitudinal research. Additionally, it is recommended that effect sizes and confidence intervals are reported along with *p*-values, as statistically significant differences are often misleading when reported alone (167), especially in underpowered studies, such as those with small sample sizes. Of the twenty longitudinal studies reviewed here, only eight provided detailed sample information, including sample size, sex distribution, and age-at-follow up visits (104, 109–111, 114–116, 121). While one study provided great detail on the sample characteristics of their longitudinal sample in their Supplementary Material, age at evaluation was not provided at the initial or follow-up visits (112). While all studies provided an approximate time interval between injury and MRI for the initial and follow-up visits, five studies did not specify details regarding average time-since-injury intervals for their pediatric TBI samples at the initial and/or follow-up visits (103–105, 111, 117). Finally, half of the studies reviewed presently included some measure of effect size with their results (103, 104, 106–108, 112–114, 117, 119).

It is important to note that, while these methodological considerations are meant to address areas that could be improved in future research, the studies included in this review are the first and only to address longitudinal outcome after pediatric TBI from a neuroimaging perspective and must be applauded for doing so. Furthermore, all of the studies reviewed here that included clinical assessments of neuropsychological or functional outcome included one or more measures recommended as basic or supplemental Common Data Elements (CDEs) by the Pediatric TBI Outcomes Working Group [see (168)]. The working groups within the TBI CDE project also suggest standardized reporting of MR image acquisition parameters (169), and all but four studies provided sufficient detail in this regard (refer to Supplementary Material for details).

## Gaps in the Literature

As reviewed in this paper, there have been a small number of studies published to date using neuroimaging to examine longitudinal changes after TBI in pediatric patients. Existing studies generally reveal dynamic changes in the months and years post-injury, but additional studies are needed to more comprehensively examine factors that may influence outcome. Severity plays an important role in outcome prediction but does not fully explain outcome heterogeneity. Additionally, the longitudinal neuroimaging studies that have been published to date are limited to investigations of pediatric TBI populations with injuries sustained no earlier than the early childhood stage of development (ages 4–6), which is likely due to difficulties associated with scanning infants and toddlers. The literature would greatly benefit from investigations of children who sustained accidental injuries at younger ages, however, and current efforts to develop multi-step procedures that ensure the comfort of young children in the MRI environment [see (170)]. The body of literature in this area is small but becomes even smaller when we consider how many individual cohorts have been examined: the tables included in this review indicate that 20 articles on 13 longitudinal cohorts have been published to date. There are a number of gaps in the literature that we hope will be addressed in the coming years.

Small sample size is the primary limitation of most neuroimaging studies of pediatric TBI. This substantially limits the ability of researchers to identify potential moderators of outcome. While the literature reviewed here suggests an effect of factors such as age, sex, and SES on outcome, these need to be examined in larger cohorts for reliability, and sources of attrition bias need to be carefully examined, disclosed and corrected for, particularly where there is a loss-to-follow up rate >20% (163, 171) or where the follow-up period is particularly long, as a relation between attrition and length of the study follow-up has been demonstrated (163). Large samples will allow for machine learning approaches to cluster demographic, clinical, and imaging variables and may reveal sub-populations within the larger patient population. There may be patterns of brain structural and functional disruption that are associated with particular cognitive, psychological, or somatic complaints. This has important implications for treatment and may help identify patients in need of more targeted treatment. The large amount of unexplained heterogeneity in post-injury outcome is a key gap in the field. There are also important outcomes that are relatively unexplored. Secondary psychiatric disorders are common post-injury but there have been very few investigations linking these to altered brain structure and function (172).

It is important to note that what constitutes an optimal comparison group for children with TBI remains an area of controversy within the field. While samples of healthy, typically-developing children are the most frequently utilized comparison group, some have argued that the use of such a comparison group fails to account for TBI-related risk factors, including predisposing neurobehavioral characteristics, such as attention-deficit/hyperactivity disorder (173) and associated impulsivity, risk-taking behavior, and substance use (174), or non-specific effects of traumatic injury, like posttraumatic stress (175, 176). Additionally, factors that may influence cognitive and functional assessment, including stress, pain, and medication effects, as well as prolonged absences from school are also not well-accounted for in TBI-related studies that use healthy comparison groups. Alternatively, other pediatric TBI studies have included children with extra-cranial or orthopedic injuries as a comparison group to account for some of the factors associated with the use of healthy children. In a recent DTI study of adolescents and young adults with mTBI (177), both typically-developing and orthopedically injured persons were included in comparison groups. Interestingly, the results of this study revealed that, relative to the typically-developing comparison group, both of the traumatically injured patient groups demonstrated similar patterns of altered white matter integrity at subacute and chronic post-injury periods, regardless of whether the injuries sustained occurred to the head. While acknowledging the strengths and limitations of each group, the authors conclude that the selection of a single comparison group may contribute to the inconsistency in dMRI findings reported in the literature. Wilde et al. (177) suggest that conclusions drawn from studies utilizing a typically-developing comparison group may be different if the studies had instead included an orthopedically-injured comparison group and therefore recommend the use of both comparison groups, if possible; however, further investigation of this issue is clearly warranted.

Advanced imaging methods and multi-modal approaches have the potential to yield important new information. Diffusion MRI is one of the most commonly used modalities in TBI neuroimaging studies, but DTI has a number of limitations. Crossing fibers can lead to inaccurate diffusion calculations when a single tensor model is used. Higher angular resolution partially addresses this, but more advanced modeling allowing for multiple fiber orientations within a voxel is also necessary. Multi-shell diffusion MRI sequences permits researchers to model both intracellular and extracellular diffusion, leading to more accurate modeling and allows for measurements of neurite density and orientation dispersion (178).

## Conclusions

**Figure 7:**
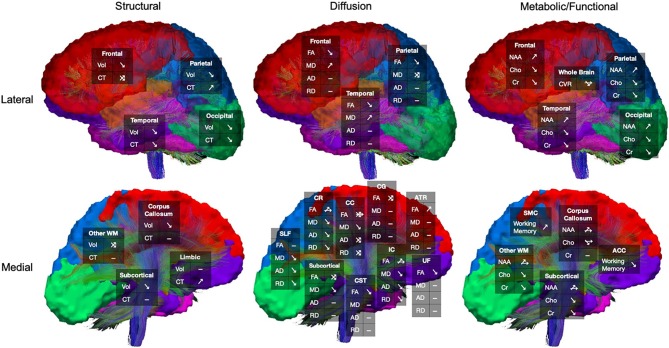
Summary of longitudinal changes in pediatric traumatic brain injury (TBI) across magnetic resonance-based neuroimaging modalities. Results are organized according to general brain region or white matter pathway. Arrows reflect changes in TBI group over time (increase, decrease, or no change). Mixed changes across the TBI group, or mixed results reported across studies, are reflected in crossed arrows. Dashes indicate no reported differences. ACC, anterior cingulate cortex; AD, axial diffusivity; ATR, anterior thalamic radiation; CC, corpus callosum; CG, cingulum; Cho, choline; CR, corona radiata; Cr, creatine; CST, corticospinal tract; CT, cortical thickness; FA, fractional anisotropy; IC, internal capsule; MD, mean diffusivity (includes ADC results); NAA, *N*-acetyl aspartate; RD, radial diffusivity; SLF, superior longitudinal fasciculus; SMC, sensorimotor cortex; TBI, traumatic brain injury; UF, uncinate fasciculus; Vol, volume; WM, white matter.

Here we review longitudinal neuroimaging studies of pediatric traumatic brain injury. See Figure 7 for a summary of the results of all longitudinal neuroimaging studies. While there is considerable heterogeneity in post-injury outcome, the literature consistently shows that alterations in brain structure, function, and metabolism can persist for an extended period of time post-injury. Longitudinal studies are particularly important for assessing changes in a developing sample, but small sample sizes have limited most studies to date. With larger sample sizes and multi-site cooperation, future studies will be able to examine potential moderators of outcome, such as the quality of the pre-injury environment, and may identify clinically meaningful patient subtypes.

## Author Contributions

HL, EW, KC, and ED contributed the conception and design of the review. HL reviewed the literature for relevant articles to include and wrote the first draft of the manuscript. EW, ED, and KC wrote sections of the manuscript. All authors contributed to the revision of the manuscript, and all authors read and approved the final submitted version.

### Conflict of Interest

The authors declare that the research was conducted in the absence of any commercial or financial relationships that could be construed as a potential conflict of interest.

## Abbreviations

AD: axial diffusivity
ADC: apparent diffusion coefficient
Cho: choline
Cr: creatine
CVR: cerebrovascular responsiveness
DAI: diffuse axonal injury
dMRI: diffusion-weighted magnetic resonance imaging
DTI: diffusion tensor imaging
FA: fractional anisotropy
fMRI: functional magnetic resonance imaging
IHTT: inter-hemispheric transfer time
MD: mean diffusivity
MRI: magnetic resonance imaging
MRS: magnetic resonance spectroscopy
msTBI: moderate-to-severe traumatic brain injury
mTBI: mild traumatic brain injury
NAA: N-acetyl aspartate
RD: radial diffusivity
ROI: region-of-interest
SES: socioeconomic status
sMRI: structural magnetic resonance imaging
SRC: sport-related concussion
TBI: traumatic brain injury
TBSS: tract-based spatial statistics.
