# Supplementary material for: Longitudinal Neuroimaging in Pediatric Traumatic Brain Injury: Current State and Consideration of Factors That Influence Recovery
Source: Front Neurol. 2019 Dec 13;10:1296. doi: 10.3389/fneur.2019.01296 (PMC6927298; doi:10.3389/fneur.2019.01296)
Supplement: Supplementary file 1 [file Data_Sheet_1.PDF]

Supplementary Table S1. Additional summary information for data extracted from structural magnetic resonance imaging studies.

Study	Race/Ethnicity	Socioeconomic Status	Mechanism of Injury	Primary Injuries	Acquisition Parameters	Functional Domains
Dennis et al., 2017b	NR	NR	14% MVA passenger<br>29% MVA pedestrian<br>57% RVA<br>5% Assault<br>5% BFT	29% Depressed SF<br>33% Non-depressed SF<br>24% SAH<br>29% SDH<br>14% IVH<br>43% EDH<br>48% ICH<br>5% DAI<br>38% Contusion<br>14% Increased ICP	3T Siemens Trio<br>3D T_1 MP-RAGE<br>TR/TE = 1900/3.26 ms<br>FOV = 250×250 mm<br>Voxel size = 1×1×1 mm<br>Flip angle = 9°	Processing speed<br>Working memory<br>Verbal learning/memory<br>Switching attention
Levin et al., 2000	NR	Parent education<br>**mmTBI:** 14.4 ± 2.4 y<br>**sTBI:** 14.0 ± 3.4 y	**mmTBI:**<br>36% High velocity<br>64% Low velocity<br><br>**sTBI:**<br>72% High velocity<br>28% Low velocity	**mmTBI:**<br>71% Diffuse injury I<br>25% Diffuse Injury II<br>4% Evacuated mass<br><br>**sTBI:**<br>20% Diffuse injury I<br>48% Diffuse injury II<br>16% Diffuse injury III<br>16% Evacuated mass	3D T_1<br>Slice thickness (T1) = 5 mm<br>Slice thickness (T2) = 1.5 mm	Outcome/Recovery<br>Adaptive Behavior
Mayer et al., 2015	NR	Parent education<br>**TBI:** 13.18 ± 2.59 y<br>**HC:** 15.62 ± 3.71 y	60% Sports/Play<br>27% Fall<br>13% MVA	None	3T Siemens<br>3D T_1 MPRAGE<br>Voxel size = 1×1×1 mm	Attention<br>Memory<br>Processing speed<br>Working memory<br>Executive function<br>Premorbid intelligence<br>Behavioral regulation<br>Emotional function
Wilde et al., 2012c	**TBI:**<br>10% African American<br>30% Caucasian<br>60% Hispanic<br><br>**OI:**<br>38% African American<br>33% Caucasian<br>28% Hispanic	SCI (*z*-score):<br>**TBI:** –0.16 ± 0.91<br>**OI:** 0.11 ± 0.82	**TBI:**<br>40% MVA passenger<br>25% RVA<br>20% Fall<br>5% MVA pedestrian<br>5% Uncategorized	NR	1.5T Philips Intera<br>3D T_1<br>TR/TE = 15/4.6 ms<br>FOV = 256×256 mm<br>Slice thickness/gap = 1/0 mm<br>Voxel size = 1×1×1 mm	Behavioral regulation<br>Emotional control
Wu et al., 2018	NR	NR	**SRC:**<br>30% Football<br>20% Running<br>10% Volleyball<br>10% Baseball<br>10% Wrestling<br>10% Other	NR	Philips<br>3D T_1 FFE<br>TR/TE = 6.9/3.1 ms<br>FOV = 256×256 mm<br>Voxel size = 1×1×1 mm<br>Flip angle = 8°	None

Wu et al., 2010	**TBI:**<br>4% African American<br>35% Caucasian<br>57% Hispanic<br>4% Native American<br><br>**OI:**<br>28% African American<br>4% Asian<br>40% Caucasian<br>24% Hispanic<br>4% Multiracial	<u>SCI (*z*-score)</u><br>**TBI:** 0.05 ± 0.91<br>**OI:** 0.27 ± 0.89<br><br><u>Maternal education</u><br>**TBI:** 12.5 ± 3.4 y<br>**OI:** 14.0 ± 2.7 y	**TBI:**<br>70% MVA passenger<br>13% RVA<br>9% MVA pedestrian<br>9% Fall	NR	1.5T Philips Intera<br>3D T_1<br>TR/TE = 15/4.6 ms<br>FOV = 256×256 mm<br>Slice thickness/gap = 1/0 mm<br>Voxel size = 1×1×1 mm	Processing speed

BFT = blunt force trauma; DAI = diffuse axonal injury; EDH = epidural hematoma; FFE = fast field echo; FOV = field of view; HC = healthy controls; ICH = intracranial hemorrhage; ICP = intracranial pressure; IVH = intraventricular hemorrhage; mmTBI = mild/moderate traumatic brain injury; MP-RAGE = magnetization prepared-rapid gradient echo; MVA = motor vehicle accident; NR = not reported; OI = orthopedically injured controls; RVA = recreational vehicle accident; SAH = subarachnoid hemorrhage; SCI = sociocomposite index (Yeates et al., 1997); SDH = subdural hemorrhage; SF = skull fracture; SRC = sports-related concussion; sTBI = severe traumatic brain injury; T = tesla; T_1 = T_1-weighted contrast; T1 = time 1; T2 = time 2; TBI = traumatic brain injury; TE = echo time; TR = repetition time

Supplementary Table S2. Additional summary information for data extracted from diffusion-weighted magnetic resonance imaging studies.

Study	Race/Ethnicity	Socioeconomic Status	Mechanism of Injury	Primary Injuries	Acquisition Parameters	Functional Domains
Dennis et al., 2018a	NR	NR	21% MVA passenger<br>25% MVA pedestrian<br>36% RVA<br>7% Assault<br>4% Fall from height<br>4% BFT<br>4% Unknown	36% Depressed SF<br>32% Non-depressed SF<br>28% SAH<br>39% SDH<br>18% IVH<br>28% EDH<br>50% ICH<br>18% DAI<br>25% Contusion<br>14% Increased ICP	3T Siemens Trio<br>DWI<br>TR/TE = 9500/87 ms<br>FOV = 256×256 mm<br>Voxel size = 2×2×2 mm<br>64 diffusion directions<br>b-values = 0/1000 s/mm^2	Processing speed<br>Working memory<br>Verbal learning/memory<br>Switching attention
Dennis et al., 2017c	NR	NR	14% MVA passenger<br>24% MVA pedestrian<br>57% RVA<br>5% Assault<br>5% BFT	30% Depressed SF<br>35% Non-depressed SF<br>30% SAH<br>30% SDH<br>15% IVH<br>45% EDH<br>50% ICH<br>5% DAI<br>40% Contusion<br>15% Increased ICP	3T Siemens Trio<br>DWI<br>TR/TE = 9500/87 ms<br>FOV = 256×256 mm<br>Voxel size = 2×2×2 mm<br>64 diffusion directions<br>b-values = 0/1000 s/mm^2	Processing speed<br>Working memory<br>Verbal memory<br>Switching attention
Ewing-Cobbs et al., 2016	**TBI:**<br>25% African American<br>31% Caucasian<br>31% Hispanic<br>13% Other<br><br>**OI:**<br>28% African American<br>28% Caucasian<br>39% Hispanic<br>5% Other	<u>Maternal education</u><br>**TBI:**<br>31% < High School<br>31% High School grad<br>19% Some college<br>19% ≥ College grad<br><br>**OI:**<br>17% < High School<br>33% High school grad<br>28% Some college<br>22% ≥ College grad	56% MVA passenger<br>19% MVA pedestrian<br>13% Fall<br>6% Sports/play<br>6% Other	<u>**Total sample** (N = 44):</u><br>(Unilateral/Bilateral/Midline)<br>14%/30%/0% Contusion<br>0%/9%/0% ICH<br>2%/16%/2% DAI<br>7%/11%/0% Edema<br>14%/18%/0% SAH/IVH<br>12%/5%/9% SDH<br>12%/7%/0% EDH	3T Philips<br>DTI EPI<br>TR/TE = 6100/84 ms<br>FOV = 240×240 mm<br>Slice thickness = 3 mm<br>Voxel size = 0.94×0.94×3 mm<br>21 diffusion directions<br>b-values = 0/1000 s/mm^2	None
Genc et al., 2017	NR	<u>AUSIE06</u><br>**TBI:** 64.85 ± 22.85<br>**HC:** 78.49 ± 11.32	82% Fall/BFT<br>18% MVA	38% Frontal lesion<br>24% Extrafrontal lesion<br>6% Subcortical lesion	3T Siemens TIM Trio<br>DTI EPI<br>TR/TE = 9300/104 ms<br>FOV = 256×256 mm<br>Slice thickness = 2 mm<br>Voxel size = 2×2×2 mm<br>60 diffusion directions<br>b-values = 0/2000 s/mm^2	Intellectual ability<br>Working memory<br>Processing speed

Mayer et al., 2015	NR	<u>Parent education</u><br>**TBI:** 13.18 ± 2.59 y<br>**HC:** 15.62 ± 3.71 y	60% Sports/Play<br>27% Fall<br>13% MVA	None	3T Siemens<br>DWI SE<br>Voxel size = 2×2×2 mm<br>30 diffusion directions<br>b-values = 0/800 s/mm^2	Attention<br>Memory<br>Processing speed<br>Working memory<br>Executive function<br>Premorbid intelligence<br>Behavioral regulation<br>Emotional function
Mayer et al., 2012	NR	<u>Parent education</u><br>**TBI:** 13.18 ± 2.59 y<br>**HC:** 15.62 ± 3.71 y	60% Sports/Play<br>27% Fall<br>13% MVA	None	3T Siemens<br>DWI SE<br>Voxel size = 2×2×2 mm<br>30 diffusion directions<br>b-values = 0/800 s/mm^2	Attention<br>Memory<br>Processing speed<br>Working memory<br>Executive function<br>Premorbid intelligence<br>Behavioral regulation<br>Emotional function
Van Beek et al., 2015	NR	NR	45% Fall<br>20% MVA passenger<br>20% MVA pedestrian<br>5% Unknown	NR	3T Philips Achieva<br>DTI EPI<br>TR/TE = 11043/55 ms<br>FOV = 220×220 mm<br>Slice thickness = 2.2 mm<br>Voxel size = 1.96×1.96×2.2 mm<br>45 diffusion directions<br>b-values = 0/800 s/mm^2	Intellectual function<br>Pre-morbid math ability<br>Numerical processing<br>Enumeration<br>Arithmetic<br>Working memory<br>Motor Speed
Verhelst et al., 2019	NR	NR	75% MVA<br>19% Sports/Play<br>6% Other	81% Frontal<br>88% Temporal<br>63% Parietal<br>50% Occipital<br>56% Corpus Callosum<br>44% Subcortical<br>31% Cerebellar<br>13% Midbrain<br>19% Brainstem	3T Siemens MAGNETOM Trio<br>DWI EPI<br>TR/TE = 10800/83 ms<br>FOV = 240×240 mm<br>Voxel size = 2.5×2.5×2.5 mm<br>64 diffusion directions<br>b-values = 0/1200 s/mm^2	Selective attention<br>Processing speed<br>Working memory<br>Nonverbal learning<br>Planning<br>Problem solving<br>Response inhibition<br>Executive function
Wilde et al., 2012a	**TBI:**<br>10% African American<br>30% Caucasian<br>60% Hispanic<br><br>**OI:**<br>38% African American<br>33% Caucasian<br>28% Hispanic	<u>SCI (z-score)</u><br>**TBI:** –0.16 ± 0.91<br>**OI:** 0.11 ± 0.82	60% MVA passenger<br>20% Fall<br>10% RVA<br>5% MVA pedestrian<br>5% Other	95% Frontal<br>60% Temporal<br>55% Parietal<br>30% Corpus Callosum<br>20% Basal Ganglia<br>15% Cerebellar	1.5T Philips Intera OR Achieva<br>DTI EPI<br>TR/TE = 6318/51 ms<br>FOV = 256×256 mm<br>15 diffusion directions<br>b-values = 0/860 s/mm^2	None

Wu et al., 2018	NR	<u>SCI</u><br>(collected, but NR)	30% Football<br>20% Running<br>10% Volleyball<br>10% Baseball<br>10% Wrestling<br>10% Other	NR	Philips<br>DTI EPI<br>TR/TE = 63180/51 ms<br>FOV = 240×240 mm<br>Slice Thickness/gap = 2/0 mm<br>Voxel size = 2×2×2 mm<br>30 diffusion directions<br>b-values = 0/1000 s/mm^2	None
Wu et al., 2010	**TBI:**<br>4% African American<br>35% Caucasian<br>57% Hispanic<br>4% Native American<br><br>**OI:**<br>28% African American<br>4% Asian<br>40% Caucasian<br>24% Hispanic<br>4% Other	<u>SCI (*z*-score)</u><br>**TBI:** 0.05 ± 0.91<br>**OI:** 0.27 ± 0.89<br><br><u>Maternal education</u><br>**TBI:** 12.5 ± 3.4 y<br>**OI:** 14.0 ± 2.7 y	70% MVA passenger<br>13% RVA<br>9% MVA pedestrian<br>9% Fall	NR	1.5T Philips Intera<br>DTI EPI<br>TR/TE = 10150.5/90 ms<br>FOV = 256×256 mm<br>Slice thickness/gap = 2.7/0 mm<br>Voxel size = 2.69×2.69×2.7 mm<br>15 diffusion directions<br>b-values = 0/860 s/mm^2	Processing speed
Yuan et al., 2017	**TBI:**<br>71% White<br>29% Non-white<br><br>**HC:**<br>91% White<br>9% Non-white	NR	NR	NR	3T Philips Achieva<br>DTI EPI<br>TR/TE = 9000/84 ms<br>FOV = 256×256 mm<br>Slice thickness = 2 mm<br>Voxel size = 2×2×2 mm<br>61 diffusion directions<br>b-values = 0/1000 s/mm^2	Attention<br>Executive function

AUSIE06 = Australian Socioeconomic Index 2006 (McMillan et al., 2009); BFT = blunt force trauma; DAI = diffuse axonal injury; DTI = diffusion tensor imaging; DWI = diffusion weighted imaging; EDH = epidural hematoma; EPI = echo planar imaging; FOV = field of view; HC = healthy controls; ICH = intracranial hemorrhage; ICP = intracranial pressure; IVH = intraventricular hemorrhage; MVA = motor vehicle accident; NR = not reported; OI = orthopedically injured controls; RVA = recreational vehicle accident; SAH = subarachnoid hemorrhage; SCI = sociocomposite index (Yeates et al., 1997); SDH = subdural hematoma; SE = single-shot echo; SF = skull fracture; T = tesla; TBI = traumatic brain injury; TE = echo time; TR = repetition time

Supplementary Table S3. Additional summary information for data extracted from magnetic resonance spectroscopy imaging studies.

Study	Race/Ethnicity	Socioeconomic Status	Mechanism of Injury	Primary Injuries	Acquisition Parameters	Functional Domains
Babikian et al., 2018	NR	Parent education<br>**TBI-Slow:** 13.0 ± 3.7 y<br>**TBI-Normal:** 14.4 ± 3.7 y<br>**HC:** 15.1 ± 3.6 y	NR	NR	3T Siemens Trio<br>3D ^{1}H EPSI<br>TR/TE = 1710/70 ms<br>Slab thickness = 135 mm<br>Spatial sampling = 100×50×18<br>FOV = 280×280×180 mm	Processing speed<br>Working memory<br>Verbal memory<br>Switching attention
Dennis et al., 2018a	NR	NR	21% MVA passenger<br>25% MVA pedestrian<br>36% RVA<br>7% Assault<br>4% Fall from height<br>4% BFT<br>4% Unknown	36% Depressed SF<br>32% Non-depressed SF<br>28% SAH<br>39% SDH<br>18% IVH<br>28% EDH<br>50% ICH<br>18% DAI<br>25% Contusion<br>14% Increased ICP	3T Siemens Trio<br>3D ^{1}H EPSI<br>TR/TE = 1710/70 ms<br>Slab thickness = 135 mm<br>Spatial sampling = 50×50×18<br>FOV = 280×280×180 mm<br>1000 spectral points	Processing speed<br>Working memory<br>Verbal memory<br>Switching attention
Holshouser et al., 2019	NR	NR	**mmTBI:**<br>19% MVA passenger<br>19% MVA pedestrian<br>6% RVA<br>34% Fall<br>19% Sports/Play<br>3% Assault<br><br>**sTBI:**<br>31% MVA passenger<br>47% MVA pedestrian<br>13% RVA<br>9% Fall	NR	3T Siemens TIM Trio<br>3D ^{1}H PRESS<br>TR/TE = 1700/144 ms<br>Slab thickness = 10 mm<br>Spatial sampling = 16×16×8<br>FOV = 160×160×80 mm<br>1024 spectral points	Intellectual ability<br>Attention<br>Memory<br>Neurological outcome
Yeo et al., 2006	NR	NR	NR	42% Hemorrhage	1.5T GE Signa<br>3D ^{1}H PRESS<br>TR/TE = 1500/62 ms<br>Slab thickness = 15 mm	Attention<br>Processing speed<br>Working memory<br>Verbal Fluency<br>Verbal Memory<br>Nonverbal Memory<br>Executive Function<br>Motor Skill

^{1}H = hydrogen; BFT = blunt force trauma; DAI = diffuse axonal injury; EDH = epidural hematoma; EPSI = echo planar spectroscopic imaging; FOV = field of view; HC = healthy controls; ICH = intracranial hemorrhage; ICP = intracranial pressure; IVH = intraventricular hemorrhage; mmTBI = mild/moderate traumatic brain injury; MVA = motor vehicle accident; NR = not reported; PRESS = point resolved spectroscopy sequence ; RVA = recreational vehicle accident; SAH = subarachnoid hemorrhage; SDH = subdural hematoma; SF = skull fracture; sTBI = severe traumatic brain injury; T = tesla; TBI-Normal = traumatic brain injury with normal interhemispheric transfer time; TBI-Slow = traumatic brain injury with slow interhemispheric transfer time; TE = echo time; TR = repetition time

Supplementary Table S4. Additional summary information for data extracted from functional magnetic resonance imaging studies.

Study	Race/Ethnicity	Socioeconomic Status	Mechanism of Injury	Primary Injuries	Acquisition Parameters	Functional Domains
Cazalis et al., 2011	NR	NR	NR	100% DAI	3T Siemens Allegra<br>EPI BOLD<br>TR/TE = 2000/30 ms<br>FOV = 200×200 mm<br>Voxel size = 3.125×3.125×3 mm<br><br>3D T_2* Flash<br>TR/TE = 57/20 ms<br>FOV = 256×256 mm<br>Voxel size = 1×0.5×2 mm<br>Flip angle = 20°	Spatial Working Memory (fMRI paradigm)
Mutch et al., 2016	NR	NR	50% Football<br>33% Hockey<br>17% Cycling<br>17% Soccer	None	3T Siemens Verio<br>T_2* GRE<br>TR/TE = 2000/30 ms<br>FOV = 240×240 mm<br>Slice thickness/gap = 5/2 mm<br>Voxel size = 3.75×3.75×6 mm<br>Flip angle = 85°	NR

BOLD = blood oxygen level dependent; DAI = diffuse axonal injury; EPI = echo planar imaging; fMRI = functional magnetic resonance imaging; FOV = field of view; GRE = gradient-recalled echo; NR = not reported; T = tesla; T_2* = T_2-weighted gradient recalled echo; TE = echo time; TR = repetition time

References

Babikian, T., Alger, J.R., Ellis-Blied, M.U., Giza, C.C., Dennis, E., Olsen, A., et al. (2018). Whole Brain Magnetic Resonance Spectroscopic Determinants of Functional Outcomes in Pediatric Moderate/Severe Traumatic Brain Injury. *J Neurotrauma* 35(14), 1637-1645. doi: 10.1089/neu.2017.5366.

Cazalis, F., Babikian, T., Giza, C., Copeland, S., Hovda, D., and Asarnow, R.F. (2011). Pivotal role of anterior cingulate cortex in working memory after traumatic brain injury in youth. *Front Neurol* 1, 158. doi: 10.3389/fneur.2010.00158.

Dennis, E.L., Babikian, T., Alger, J., Rashid, F., Villalon-Reina, J.E., Jin, Y., et al. (2018a). Magnetic resonance spectroscopy of fiber tracts in children with traumatic brain injury: A combined MRS - Diffusion MRI study. *Hum Brain Mapp*. doi: 10.1002/hbm.24209.

Dennis, E.L., Faskowitz, J., Rashid, F., Babikian, T., Mink, R., Babbitt, C., et al. (2017b). Diverging volumetric trajectories following pediatric traumatic brain injury. *Neuroimage Clin* 15, 125-135. doi: 10.1016/j.nicl.2017.03.014.

Dennis, E.L., Rashid, F., Ellis, M.U., Babikian, T., Vlasova, R.M., Villalon-Reina, J.E., et al. (2017c). Diverging white matter trajectories in children after traumatic brain injury: The RAPBI study. *Neurology* 88(15), 1392-1399. doi: 10.1212/wnl.0000000000003808.

Ewing-Cobbs, L., Johnson, C.P., Juranek, J., DeMaster, D., Prasad, M., Duque, G., et al. (2016). Longitudinal diffusion tensor imaging after pediatric traumatic brain injury: Impact of age at injury and time since injury on pathway integrity. *Hum Brain Mapp* 37(11), 3929-3945. doi: 10.1002/hbm.23286.

Genc, S., Anderson, V., Ryan, N.P., Malpas, C.B., Catroppa, C., Beauchamp, M.H., et al. (2017). Recovery of White Matter following Pediatric Traumatic Brain Injury Depends on Injury Severity. *J Neurotrauma* 34(4), 798-806. doi: 10.1089/neu.2016.4584.

Holshouser, B., Pivonka-Jones, J., Nichols, J.G., Oyoyo, U., Tong, K., Ghosh, N., et al. (2019). Longitudinal Metabolite Changes after Traumatic Brain Injury: A Prospective Pediatric Magnetic Resonance Spectroscopic Imaging Study. *J Neurotrauma* 36(8), 1352-1360. doi: 10.1089/neu.2018.5919.

Levin, H.S., Benavidez, D.A., Verger-Maestre, K., Perachio, N., Song, J., Mendelsohn, D.B., et al. (2000). Reduction of corpus callosum growth after severe traumatic brain injury in children. *Neurology* 54(3), 647-653. doi: 10.1212/wnl.54.3.647.

Mayer, A.R., Hanlon, F.M., and Ling, J.M. (2015). Gray matter abnormalities in pediatric mild traumatic brain injury. *J Neurotrauma* 32(10), 723-730. doi: 10.1089/neu.2014.3534.

Mayer, A.R., Ling, J.M., Yang, Z., Pena, A., Yeo, R.A., and Klimaj, S. (2012). Diffusion abnormalities in pediatric mild traumatic brain injury. *J Neurosci* 32(50), 17961-17969. doi: 10.1523/jneurosci.3379-12.2012.

McMillan, J., Beavis, A., and Jones, F.L. (2009). The AUSEI06: A new socioeconomic index for Australia. *Journal of Sociology* 45(2), 123-149.

Mutch, W.A., Ellis, M.J., Ryner, L.N., Morissette, M.P., Pries, P.J., Dufault, B., et al. (2016). Longitudinal Brain Magnetic Resonance Imaging CO2 Stress Testing in Individual Adolescent Sports-Related Concussion Patients: A Pilot Study. *Front Neurol* 7, 107. doi: 10.3389/fneur.2016.00107.

Van Beek, L., Vanderauwera, J., Ghesquiere, P., Lagae, L., and De Smedt, B. (2015). Longitudinal changes in mathematical abilities and white matter following paediatric mild traumatic brain injury. *Brain Inj* 29(13-14), 1701-1710. doi: 10.3109/02699052.2015.1075172.

Verhelst, H., Giraldo, D., Vander Linden, C., Vingerhoets, G., Jeurissen, B., and Caeyenberghs, K. (2019). Cognitive Training in Young Patients With Traumatic Brain Injury: A Fixel-Based Analysis. *Neurorehabil Neural Repair*, 1545968319868720. doi: 10.1177/1545968319868720.

Wilde, E.A., Ayoub, K.W., Bigler, E.D., Chu, Z.D., Hunter, J.V., Wu, T.C., et al. (2012a). Diffusion tensor imaging in moderate-to-severe pediatric traumatic brain injury: changes within an 18 month post-injury interval. *Brain Imaging Behav* 6(3), 404-416. doi: 10.1007/s11682-012-9150-y.

Wilde, E.A., Merkley, T.L., Bigler, E.D., Max, J.E., Schmidt, A.T., Ayoub, K.W., et al. (2012c). Longitudinal changes in cortical thickness in children after traumatic brain injury and their relation to behavioral regulation and emotional control. *Int J Dev Neurosci* 30(3), 267-276. doi: 10.1016/j.ijdevneu.2012.01.003.

Wu, T., Merkley, T.L., Wilde, E.A., Barnes, A., Li, X., Chu, Z.D., et al. (2018). A preliminary report of cerebral white matter microstructural changes associated with adolescent sports concussion acutely and subacutely using diffusion tensor imaging. *Brain Imaging Behav* 12(4), 962-973. doi: 10.1007/s11682-017-9752-5.

Wu, T.C., Wilde, E.A., Bigler, E.D., Li, X., Merkley, T.L., Yallampalli, R., et al. (2010). Longitudinal changes in the corpus callosum following pediatric traumatic brain injury. *Dev Neurosci* 32(5-6), 361-373. doi: 10.1159/000317058.

Yeates, K.O., Taylor, H.G., Drotar, D., Wade, S.L., Klein, S., Stancin, T., et al. (1997). Preinjury family environment as a determinant of recovery from traumatic brain injuries in school-age children. *J Int Neuropsychol Soc* 3(6), 617-630.

Yeo, R.A., Phillips, J.P., Jung, R.E., Brown, A.J., Campbell, R.C., and Brooks, W.M. (2006). Magnetic resonance spectroscopy detects brain injury and predicts cognitive functioning in children with brain injuries. *J Neurotrauma* 23(10), 1427-1435. doi: 10.1089/neu.2006.23.1427.

Yuan, W., Treble-Barna, A., Sohlberg, M.M., Harn, B., and Wade, S.L. (2017). Changes in Structural Connectivity Following a Cognitive Intervention in Children With Traumatic Brain Injury. *Neurorehabil Neural Repair* 31(2), 190-201. doi: 10.1177/1545968316675430.
